# Prevention of polycystic ovary syndrome and postmenopausal osteoporosis by inhibiting apoptosis with Shenling Baizhu powder compound

**DOI:** 10.7717/peerj.13939

**Published:** 2022-10-28

**Authors:** Jing Liang, Ai-li Bao, Hong-yu Ma, Wei Dong, Wei-hua Li, Xi Wu, Han-yu Li, Hai-yan Hou, Ya-qiong Chen, Jia-lin Fu, Chao Shao

**Affiliations:** 1 Department of Gynecology, Guang’anmen South Area Hospital, China Academy of Chinese Medical Sciences, Beijing, China; 2 Hebei General Hospital, Department of Traditional Chinese Medicine, Hebei, Chinese; 3 Department of Obstetrics and Gynecology, Characteristic Medical Center of Chinese People’s Armed Police Force, Tianjin, China

**Keywords:** Shenling Baizhu powder compound, Aromatic hydrocarbon receptor (AHR), Polycystic ovary syndrome (POCS), Postmenopausal osteoporosis (PMO), Cell apoptosis

## Abstract

**Objective:**

Shenling Baizhu powder (SBP) has been shown to reverse the abnormal expression of the aromatic hydrocarbon receptor (AHR) mediated by air pollution. Our study aimed to understand the main ingredient of SBP and investigate its action mechanism in preventing polycystic ovary syndrome (POCS) and postmenopausal osteoporosis (PMO).

**Methods:**

The active ingredients of SBP with the highest binding affinity to AHR were screened using a Chinese medicine database, and their binding mechanism was simulated using molecular dynamics simulation (MDS). Rutin was utilized to treat ovarian granulosa cell lines and osteoblast cell lines. The cell lines were treated with a gradient of rutin concentration (0.01 mmol/L, 0.05 mmol/L and 0.1 mmol/L) to find the optimal drug dose. PCR was used to detect AHR and apoptosis-related proteins, and WB to detect the expression of AHR, caspase-3 and cleaved-caspase-3. Finally, the CCK-8 cell proliferation assay detected the proliferation of cells.

**Results:**

We obtained Rutin through the Chinese medicine database, and dynamics simulation determined its binding sites. Ovarian granulosa cell lines and osteoblast cell lines were treated with Rutin. RT-PCR and western blotting revealed that the expression of apoptosis-associated protein Bcl-2 was elevated, and the expression of AHR, Bax, caspase-3 and PARP were decreased. CCK-8 results showed accelerated proliferation in both cell types.

**Conclusion:**

Rutin, the main ingredient of SBP compound, works by binding to AHR, which can improve POCS and PMO by inhibiting cell apoptosis and by promoting cell proliferation.

## Introduction

With modern-day industrialization and urbanization, inhalable particulate matter (PM2.5 and PM10), SO2, NO2, and polycyclic aromatic hydrocarbons are released, sharply deteriorating the air quality. It also increases environmental endocrine disruptors, which promote the development of respiratory diseases, cardiovascular diseases, and endocrine diseases, and seriously affect human health (Wu, Jin & Carlsten, 2018; Schraufnagel et al., 2019; Moran et al., 2019). The aromatic hydrocarbon receptor (AHR) binds to endocrine-disrupting chemicals and participates in the regulation of cellular homeostasis. It plays an essential role in the occurrence and development of tumors, cardiovascular diseases, endocrine diseases and other diseases (Esser et al., 2018). Studies have shown that air pollution can affect the expression of AHR. Air pollutants, such as polycyclic aromatic hydrocarbons can bind to intracellular AHR, resulting in oxidative stress damage and abnormal immune regulation (Vogel et al., 2020). Moreover, some studies have pointed out that environmental endocrine disruptors such as polycyclic aromatic hydrocarbons may affect reproductive endocrine function through the AHR signalling pathway and participate in the occurrence of reproductive endocrine diseases such as polycystic ovary syndrome (PCOS) and postmenopausal osteoporosis (PMO) (Naruse et al., 2002; Vandenberg et al., 2009). Increasing attention is being paid to AHR dysfunction caused by air pollution.

PCOS is a common endocrine disorder in women of reproductive age. The main clinical manifestations include hyperandrogenemia, ovulation dysfunction and polycystic follicular morphology, a common cause of infertility in women. In addition to reproductive endocrine disorders, PCOS may also be accompanied by insulin resistance and abnormal blood lipid metabolism (ACOG Practice Bulletin No. 194, 2020). The pathogenesis of PCOS is complex, involving epigenetic and environmental factors (Goodarzi et al., 2011). Studies have found that exposure to high concentrations of air pollutants such as NO_2_, SO_2_ and cigarette smoke can increase the risk of PCOS in women (Li et al., 2018; Merlo et al., 2019; Lin et al., 2019).

PMO is a systemic bone disease related to the decrease of estrogen level and the decline of ovarian function, mainly characterized by increased bone fragility and destruction of bone microstructure. The main clinical manifestations include increased risk of fracture, dyskinesia, and chronic pain, which seriously affect the life quality of patients and increase the risk of death (Arceo-Mendoza & Camacho, 2021). Studies have shown that some environmental factors such as a history of prolonged cigarette exposure, can also increase the risk of PMO, suggesting that environmental pollution promotes the occurrence of PCOS and PMO (Banjabi et al., 2022). Interestingly, studies have found that serum AHR exogenous ligand levels are significantly increased in patients with PCOS (Yang et al., 2015; Rutkowska & Diamanti-Kandarakis, 2016). A combination of aromatic 3-Methylcholanthrene and AHR inhibits the proliferation and differentiation of osteocytes, suggesting that the active components of the drug might be involved in the occurrence and development of PCOS ad PMO through AHR (Naruse et al., 2002).

MDS is widely used in drug design and life sciences to study conformational changes and functional resolution of molecules (Liu et al., 2018; Chen et al., 2022; Kang et al., 2022). Combining computational simulations with experiments has become an important research tool recently because it can greatly predict, guide, and interpret experiments (Paquet & Viktor, 2015; Hildebrand, Rose & Tiemann, 2019; Lu et al., 2022). In the treatment of diseases, Chinese medicine plays an important role (Gao, Gao & Xiao, 2021; Ong, 2021; Tao et al., 2022; Zhang et al., 2022b). The traditional Chinese herbal compound Shenling Baizhu powder (SBP) is used to treat inflammation, antioxidants, immunomodulators, and to promote apoptosis (Tang et al., 2020; Wang et al., 2021; Pan et al., 2021). It has shown significant value in inflammatory diseases, endocrine diseases, tumors and other diseases (Tang et al., 2020; Feng et al., 2020). However, the effect of the SBP compound on PCOS and PMO is unclear. In this study, we explored the mechanism of SBP on PM2.5-induced abnormal AHR function, and explored its applications in PCOS and PMO by studying molecular dynamics.

## Methods

### Screening of active components of drugs

According to the name of traditional Chinese medicine, the active components of Shenling Baizhu powder were obtained from Herbal Ingredients’ Targets Platform 2.0 (HIT 2.0) (http://www.badd-cao.net:2345/) (Ye et al., 2011; Yan et al., 2022). The active components of Shenling Baizhu powder included: *Amomum villosum*, *Atractylodes macrocephala*, *Coix lacryma-jobi var. ma-yuen, Dioscorea batatas*, *Glycyrrhiza uralensis*, *Lablab purpureus*, *Nelumbo nucifera*, *Panax ginseng*, *Platycodon grandiflorum*, and *Wolfiporia cocos*. The quantum chemistry optimization of small molecules under the B3LYP/6-31G* basis set was carried out with the quantitative software Orca. The process involved correcting the dihedral angle of the bond length and calculating the fixed charge.

### Molecular docking

GeneCards was used to identify the protein structure of AHR. Using smina, the ligand with polar hydrogen and the correct protonated protein were set as box center, and then docking was started. The lowest energy conformation was selected as the final conformation to start the dynamics simulation.

### Molecular dynamics simulation (MDS)

The docking results were selected as the initial structure and amber14sb as the position. TIP3P water model was used to add solvents to the complex system. Subsequently, the water box was established, and the sodium ion equilibrium system was added. The Verlet and cg algorithms and PME were used in the elastic simulation to deal with the electrostatic interaction. The steepest descent method was used to minimize the energy of the maximum number of steps (50,000 steps). The cutoff distance of Coulomb force and van der Waals radius were both 1.4 nm. Finally, the canonical system (NVT) and isothermal isobaric system (NPT) were used to balance the system. The MD simulation of 100 ns was carried out at room temperature and pressure, and the free energy was calculated by mmpbsa.

### Cell culture

The hFOB1.19 osteoblast cell line and KGN ovarian granulosa cell line were purchased from the Shanghai Institute of Cell Research, Chinese Academy of Sciences, and cultured at 37 °C with 5% CO_2_. KGN cells were cultured in DMEM + 10% FBS + 1% double-antibody, while hFOB1.19 cells were cultured in DMEM + 10% FBS + 1% double-antibody. The cultured cells were used for follow-up treatment. The cells were treated with different concentrations of Rutin (0.01 mmol/L, 0.05 mmol/L and 0.1 mmol/L) (Selleck, Houston, TX, USA) for 48 h, followed by 100um ferrous sulfate (Sangon, Shanghai, China) and 600 um hydrogen peroxide (Sangon, China) for one hour. The cells were then collected for follow-up experiments.

### RNA extraction and RT-PCR analysis

TRIzol (Sangon, Shanghai, China) was used to obtain RNA from the osteoblast cell line and ovarian granulosa cell line treated above according to the manufacturer’s instructions as previous researches (Rio et al., 2010; Ma et al., 2022; Xuan et al., 2022). The obtained RNA was reverse transcribed into cDNA using GoScript ™ Reverse Transcription Kit (Promega, Madison, WI, SA), and the mRNA expression level was subsequently determined using FastFire qPCR PreMix (SYBR Green) (Tiangen, Beijing, China). ABI applied biosystems 7500 (Applied Biosystems, Waltham, MA, USA) was used to perform RT- PCR. The mRNA expression level of the target gene was calculated as 2^−ΔΔCt^, and GAPDH was used as the internal reference (Table S2).

### Protein extraction and western blotting

The harvested cells were subjected to protein extraction using protease inhibitors containing RIPA buffer. Equal amounts of total protein were separated by 10% SDS-PAGE gel while transferred to the PVDF membrane. The membrane was then incubated with 5% BSA at room temperature for 2 h to block non-specific signals. Primary antibody incubation was performed overnight, and secondary antibody incubation was performed at room temperature for 2 h. Developer exposure was performed, and the images were stored. The primary antibodies used above included anti-caspase-3 (1:1000; CST, Danvers, CA, USA), anti-cleaved caspase-3 (1:1000; Absin, Shanghai, China), anti AHR (1:1000; CST, Danvers CA) and anti-GAPDH (1:2000; Servicebio, Wuhan, China).

### CCK-8 cell proliferation assay

The CCK-8 assay was used to determine the proliferative capacity of osteoblasts and ovarian granulosa cells. We divided the Rutin-treated cells equally into 96-well plates and counted the cells. We added 1*10^3^ cells per well, and cell proliferation was determined using CCK-8 for five consecutive days. 10 µL of CCK-8 reagent was added to each well, and OD450 values were measured after 2.5 h and plotted for analysis.

### Statistical analysis

All experimental samples included data from at least three independent experiments, and data were expressed as mean ± standard deviation. Statistical differences between two independent samples were analyzed using the chi-square test and the *t*-test. Statistical significance was defined as *P* < 0.05.

## Results

### Rutin binds strongly to AHR

**Figure 1:**
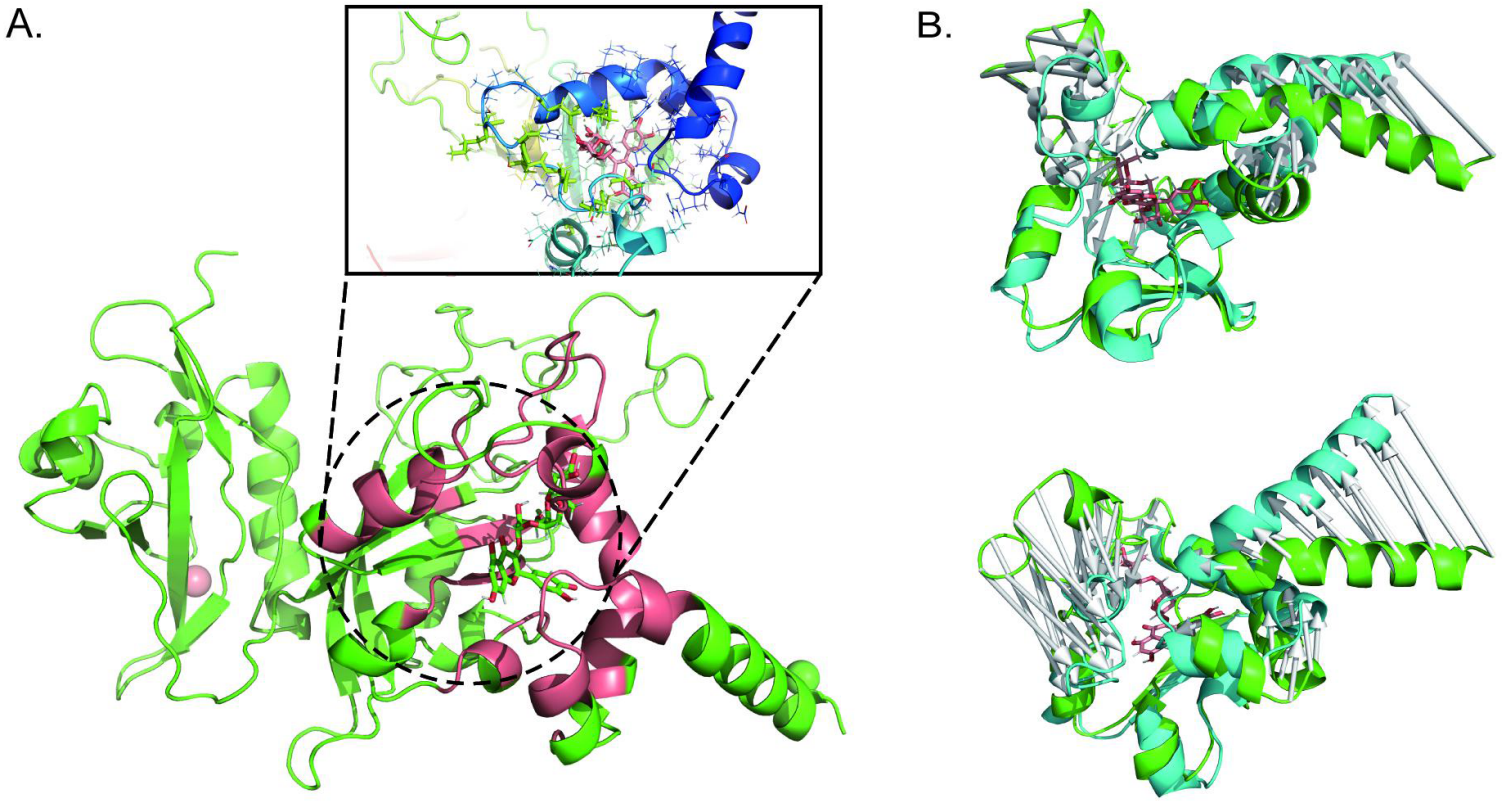
Molecular docking and molecular dynamics reveal that rutin targets binding to AhR. (A) Pattern diagram of molecular docking of rutin and AhR; (B) pattern diagram of molecular dynamics simulation changes of Rutin-AhR complex in aqueous solution, green represents before molecular dynamics simulation, cyan indicates after molecular dynamics simulation.

According to the name of traditional Chinese medicine, the active components of Shenling Baizhu powder were obtained from Herbal Ingredients’ Targets Platform 2.0 (HIT 2.0) (http://www.badd-cao.net:2345/). Active ingredients of Shenling Baizhu powder compound included: *Amomum villosum*, *Atractylodes macrocephala*, *Coix lacryma- jobi var. ma - yuen*, *Dioscorea batatas, Glycyrrhiza uralensis*, *Lablab purpureu* s, *Nelumbo nucifera, Panax ginseng*, *Platycodon grandiflorum*, and *Wolfiporia cocos*. Therefore, 256 active ingredients of the Shenling Baizhu powder compound were found after de-duplication and are listed in Table S1. Molecular docking revealed that rutin was the small molecule with the most robust binding ability to AHR (−11.1 kcal/mol, Fig. 1A).

### Molecular dynamics simulation (MDS)

**Figure 2:**
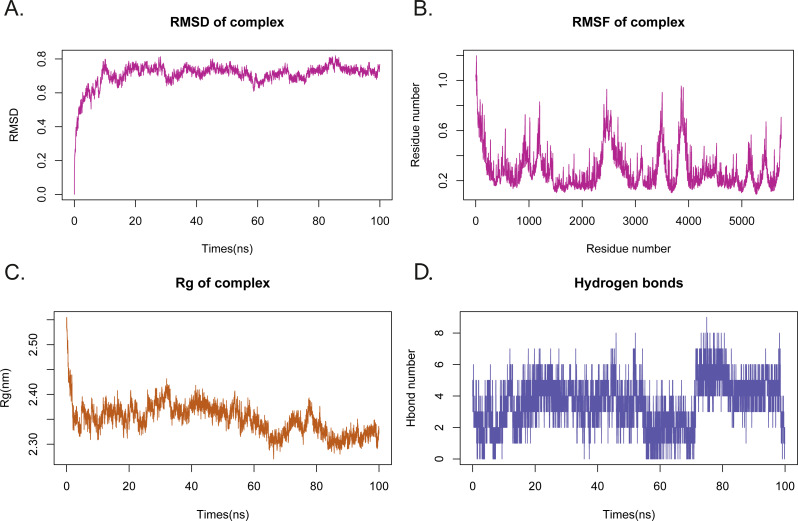
Parameter variation of molecular dynamics simulations. (A) Variation of RMSD with simulation time during molecular dynamics simulation (MDS); (B) root mean square fluctuation (RMSF) can observe the local site conformation of the system during the simulation; (C) variation of Rg with simulation time during MDS; (D) variation of hydrogen bonding with simulation time during MDS.

To investigate the stability and dynamics characteristics of the Rutin-AHR complex in an aqueous solution, we performed MD simulations (Fig. 1B). The atomic root mean square deviation (RMSD) can measure the stability of the system. RMSD values of the Rutin-AhR complex converged to 0.8 nm after the system equilibrium (after 40 ns), indicating that the protein-ligand binding orientation was good and can rapidly contribute to the overall mechanism to reach a steady-state (Fig. 2A). The root mean square fluctuation (RMSF) can observe the local site conformation of the system during the simulation (Fig. 2B). Excluding the null interference in the loop region in the vicinity of the conformational binding pocket (amino acids of sites 49-60, 72-102 and 111-120 changed dramatically during the simulation), this change consisted of the “induced fit” theory, indicating that the protein and small molecule were adjusting toward homeostasis in both directions. The radius of gyration (Rg) is an important indicator used to evaluate the tightness of the structure (Fig. 2C). Also, we found a fluctuation at 5-15 ns, corresponding to the ripple in RMSD, indicating that the system was undergoing a transition from the docked transient pseudo-positive steady-state to the sub-steady state and then to the equilibrium state, similarly at 33 ns and 45 ns. We further described the relation between the number of hydrogen bonds in the Rutin-AHR complex and simulation time, showing the role of specific recognition in ligand recognition laterally (Fig. 2D). Furthermore, it exhibited two hydrogen bonding fluctuations, one around 5-15 ns and the other after 33-45 ns.

### Binding mechanisms for MDS

**Figure 3:**
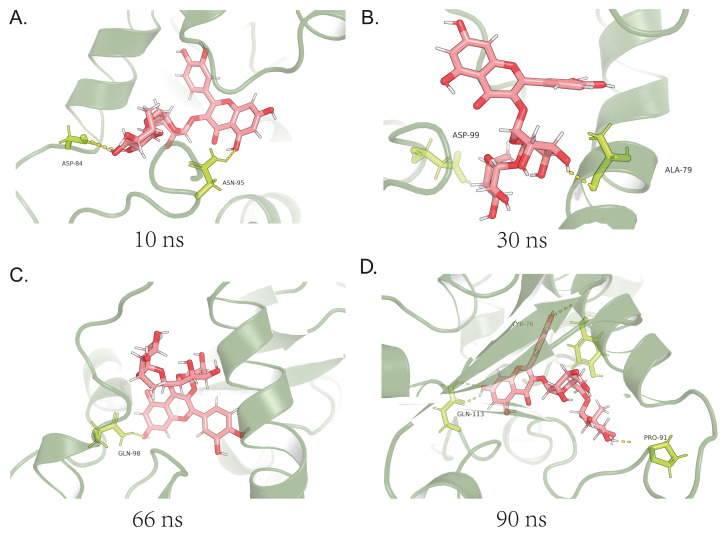
Analysis of binding mechanisms for MDS. (A) At 10 ns in MDS, there is the first fluctuation after change of the false positive state obtained by docking,and it can be seen that there is an interaction between ASP-84 and ASN-95 at this time; (B) at 30 ns, the *α*-Helix, where ALA-79 is located, is completely close to the small molecule due to the pre-adsorption of the loop region, and so is ASP-99; (C) at 66 ns in MDS, the small molecule starts to fold in the flexible section, and affected by this, only GLN-98 is connected at this moment; (D) at 90 ns in MDS, the small molecule has finished folding and reached a new equilibrium.

At 5 ns, the first fluctuation after changing the false-positive state obtained from docking, an interaction can be seen between ASP-84 and ASN-95, both acting as proton acceptors. The loop region was subjected to electrostatic adsorption and started to move inward (Fig. 3A). At 15 ns, *α*-Helix, where ALA-79 and ASP-99 were located, is close to the small molecule under the pre-loop region (Fig. 3B). At this point, two hydrogen bonds were in contact. In combination with the 5-15 ns data, it can be seen that the upper left part of the small molecule is firmly fixed deep in the pocket without change. At 33 ns, the flexible section of the small molecule started to fold over, and only GLN-98 remained connected, leaving the small molecule in a flexible state (Fig. 3C). At 45 ns, the small molecule has finished folding over and reached a new equilibrium. Finally, the three residues, TYR-76, PRO-91 and GLN-113, formed four hydrogen bonds from three angles to fix the small molecule (Fig. 3D).

### Rutin promoted cell proliferation by inhibiting granulosa cell apoptosis

**Figure 4:**
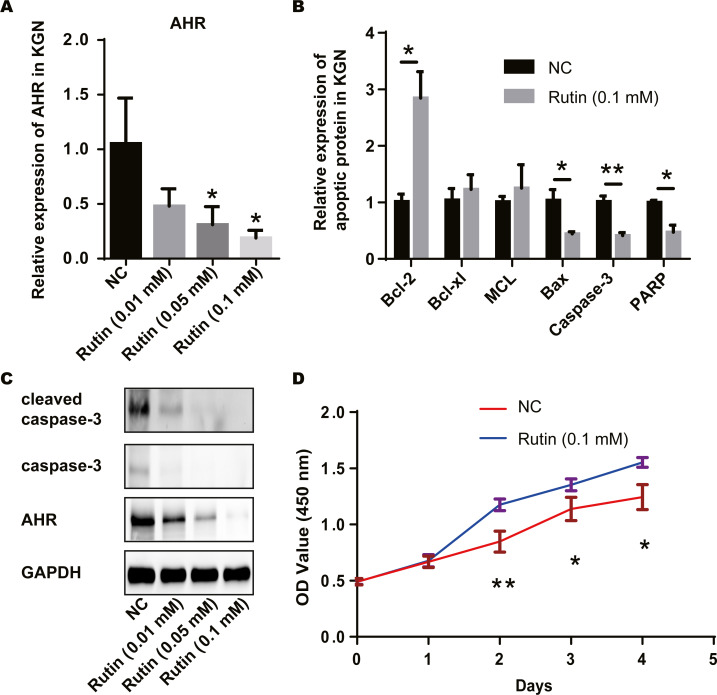
Rutin promotes cell proliferation by inhibiting apoptosis in ovarian granulosa cells. (A) RT-PCR results indicate that Rutin can reduce the expression of AHR in ovarian granulosa cells, and the expression of AHR decreases as the concentration of Rutin increases; (B) RT-PCR results show that Rutin (0.1 mM) can elevate the expression of Bcl-2 and reduce the expression of Bax, caspase-3 and PARP; (C) western blot results show that Rutin could decrease the expression of AHR, caspase-3, cleaved caspase-3; (D) CCK-8 results show that Rutin (0.1 mM) could promote the proliferation of ovarian granulosa cells. * *P* ≤ 0.05, ** *P* ≤ 0.01.

We treated the ovarian granulosa cell line with rutin and detected the expression of AHR and apoptosis-related proteins *via* RT-PCR. AHR levels decreased with the increase in Rutin concentration (Fig. 4A). Compared to the control group, the expression of apoptosis-related proteins Bcl-2 was elevated and the expression of Bax, caspase-3 and PARP were decreased in the experimental group. However, the expression of Bcl-xL and MCL did not show significant differences (Fig. 4B). Western blot shows a decrease in AHR, caspase-3 and cleaved-caspase-3 levels after Rutin treatment. Our results showed that with increasing the concentration of Rutin, the expression of these proteins decreased proportionately (Fig. 4C). And the cells proliferated faster under Rutin treatment (Fig. 4D). Therefore, we suggested that Rutin could inhibit the apoptosis of granulosa cells by suppressing the expression of AHR and promoting cell proliferation. This might be the mechanism behind the positive effect of Rutin on PCOS.

### Rutin promoted cell proliferation by inhibiting osteoblast apoptosis

**Figure 5:**
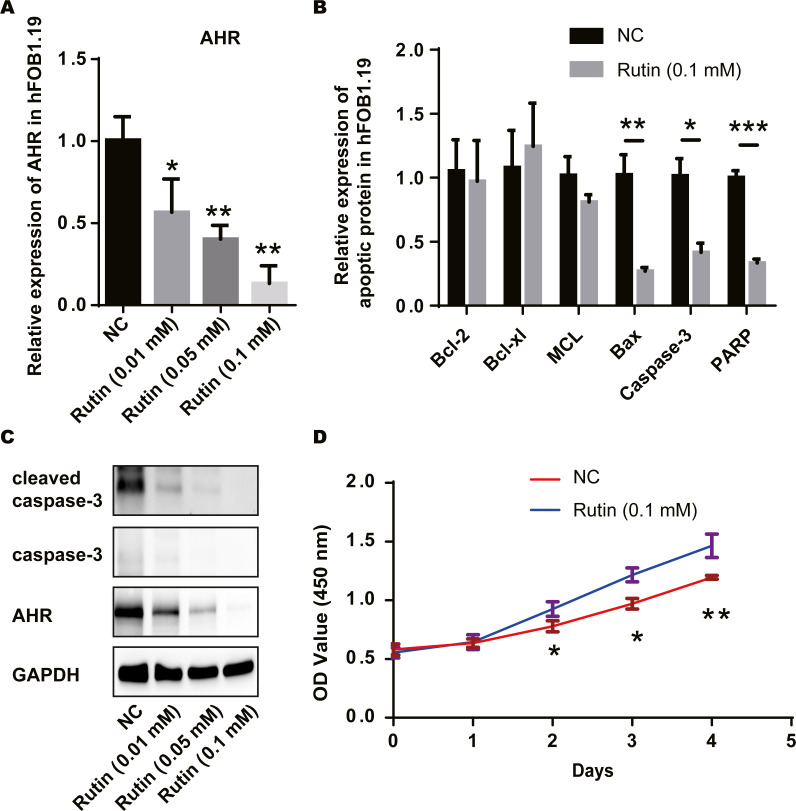
Rutin promotes cell proliferation by inhibiting apoptosis in osteoblasts. (A) RT-PCR results indicate that Rutin can reduce the expression of AHR in osteoblasts, and the expression of AHR decreases as the concentration of Rutin increases; (B) RT-PCR results show that Rutin (0.1 mM) can reduce the expression of Bax, caspase-3 and PARP; (C) Western blot results show that Rutin can reduce the expression of AHR, caspase-3 and cleaved caspase-3;D: CCK-8 results show that Rutin (0.1 mM) could promote the proliferation of osteoblasts. * *P* ≤ 0.05, ** *P* ≤ 0.01,*** *P* ≤ 0.001.

Older women are more likely to suffer from PMO (Lane, 2006; Johnston & Dagar, 2020). AHR and PMO have previously been found to be closely related, and the rutin-AHR relationship in MDS suggest that rutin may help improve PMO (Mazerska et al., 2016; Weng, Li & Zhu, 2022; Zhang et al., 2022a). We treated the osteoblast cell line with Rutin and used RT-PCR and western blot to detect apoptosis-related proteins. As the concentration of Rutin increased, the expression of AHR decreased consequently (Fig. 5A). Expression levels of apoptosis-related proteins, Bax, caspase-3 and PARP, decreased in the experimental group compared with that of the control group. Moreover, significant differences were observed in the expression of Bcl-2, Bcl-xL and MCL (Fig. 5B). Western blot showed a decrease in the expression of AHR, caspase-3, and cleaved-caspase-3 after Rutin treatment. Our results showed that with increasing the concentration of Rutin, the expression level of AHR, caspase-3 and cleaved-caspase-3 decreased proportionately (Fig. 5C). According to our results, Rutin could inhibit the apoptosis of osteoblasts and promote cell proliferation by suppressing AHR expression (Fig. 5D). This mechanism might be involved in the preventive effect of Rutin on PMO. Together, we show that Rutin in the compound of Shenling Baizhu powder can reduce the expression of AHR, reduce apoptosis protein levels, and promote cell proliferation, preventing PCOS and PMO.

## Discussion

Shenling Baizhu powder (SBP) compound can resist oxidative stress, improve lung function, regulate intestinal flora, and promote apoptosis of tumor cells. It has potential clinical application in various diseases such as chronic enteritis, chronic obstructive pulmonary disease, and tumors (Xi et al., 2016; Zhang et al., 2018; Wang et al., 2021). In this study, we found that Rutin in the SBP compound reduced the expression of AHR. PCOS and PMO may be prevented by SBP by reducing expression of apoptotic proteins and promoting cell proliferation.

Rutin is a natural antioxidant, which can effectively scavenge oxygen free radicals, regulate cell growth and differentiation, anti-inflammatory, and regulate apoptosis and autophagy (Mahendra et al., 2020). Previous studies have shown that Rutin can reduce vancomycin-induced renal tubular apoptosis by inhibiting apoptosis, reducing intracellular reactive oxygen species, and improving mitochondrial dysfunction (Qu et al., 2019). Rutin upregulates miR-877-3p expression, inhibits Bcl-2 transcription and induces apoptosis in pancreatic cancer cells (Huo et al., 2022). Rutin also inhibits autophagy and Akt activation-mediated apoptosis and attenuates adriamycin-induced cardiotoxicity (Ma et al., 2017). These studies suggest its protective effects on cardiovascular, oncological, inflammatory, and other diseases. In addition, Rutin has been shown to reduce cell apoptosis and promote activation and growth of primordial follicles by regulating PI3K/Akt signalling (Lins et al., 2021). It can regulate ovarian metabolism and hormonal disorders, improve ovarian failure, and have significant clinical value in reproductive endocrine diseases (Jahan et al., 2016; Hu et al., 2017). Rutin also has significant anti-osteoporotic activity, promoting osteoblast differentiation and preventing ovariectomy-induced PMO (Yang et al., 2018; Liu et al., 2021). Together, it suggests that Rutin has potential value in the clinical management of POCS and PMO.

Rutin is a flavonoid, and studies have shown that flavonoids can modulate or activate various nuclear receptors, including AHR (Wang et al., 2020; Liu et al., 2020). AHR is a ligand-dependent transcription factor that can be activated by binding to exogenous aromatic hydrocarbon compounds. It is expressed in various tissues and is involved in multiple biological processes, such as cell growth and differentiation, apoptosis, hormone metabolism, and stress response (Esser et al., 2018). We obtained Rutin, the active substance bound most strongly to AHR, through the Chinese medicine database and simulated the binding mechanism by MDS. We identified Rutin as a binding site for AHR. The Rutin-AHR complex exhibited a good binding degree, suggesting that Rutin may act by binding to AHR. MDS use computer technology and theoretical approaches to simulate molecular motions, and capture a variety of biomolecular processes, including conceptual changes, ligand binding, etc (Collier, Piggot & Allison, 2020). It is now widely used in biomedical research because it is not limited by test techniques and sample preparation.

Environmental pollution has led to the release of many endocrine-disrupting chemicals such as polycyclic aromatic hydrocarbons. Previous studies have shown that endocrine-disrupting chemicals can activate AHR expression and lead to abnormal AHR expression (Vogel et al., 2019). Also, air pollution can affect the expression of AHR (Vogel et al., 2020). Furthermore, abnormal AHR expression can promote the development of POCS and PMO (Wang et al., 2020). PCOS and PMO are reproductive endocrine diseases associated with endocrine disruptors through the AHR signaling pathway (Naruse et al., 2002; Vandenberg et al., 2009). By interacting with AHR, rutin, an active ingredient in SBP, may prevent these diseases caused by environmental pollution.

Traditionally, SBP is used to treat inflammation, antioxidants, immunomodulators, and to promote apoptosis (Tang et al., 2020; Wang et al., 2021; Pan et al., 2021). Inflammatory diseases, endocrine diseases, tumors, and other diseases have been demonstrated to benefit from it (Tang et al., 2020; Feng et al., 2020). Specifically, this study investigated the potency of SBP on AHR. Molecular dynamics simulations revealed that Rutin was the only active ingredient that was capable of inhibiting AHR. Furthermore, other ingredients of SBP in POCS and PMO were not evaluated in this study. Therefore, further research is needed. According to this study, rutin can inhibit granulosa cell apoptosis, promote cell proliferation, prevent ovarian destruction, and play a protective role in PCOS. Similarly, Rutin was also found to inhibit osteoblast apoptosis and promote proliferation, which regulates the balance of bone metabolism between osteoblasts and osteoclasts and inhibits the progression of PMO. These results suggested that Rutin had a protective effect on PCOS and PMO. In conclusion, this study found that Rutin, the main component of Shenling Baizhu powder compound, could bind to AHR, thus inhibiting cell apoptosis and promoting cell proliferation to improve PCOS and PMO. By downregulating AHR and apoptosis-related proteins, Rutin inhibits apoptosis in cellular experiments. Environmental pollution causes PCOS and PMO, which may be prevented by studying the regulatory mechanisms of SBP in vitro. However, there is no clear mechanism for how Rutin inhibits apoptosis by downregulating AHR. Therefore, more extensive data samples are required to validate the conclusions. There will be a need for further animal labs since the cells are not directly derived from the patient. Physiological changes resulting from POCS and PMO will need to be examined further in future studies using multiple cell lines. There should be further investigation of some osteoblastic secretions that affect its maturation since decreased OPG secretion is associated with increased osteoclastic activity after menopause.

## Conclusions

Rutin, the main component of SBP, inhibits apoptosis and promotes cell proliferation by binding to AHR. This study provides a possible therapeutic strategy for treating POCS and PMO, as well as a basis for the clinical application of SBP.

##  Supplemental Information

10.7717/peerj.13939/supp-1 Supplemental Information 1 Raw data and plot

10.7717/peerj.13939/supp-2 Supplemental Information 2 All the active ingredients of Shenling Baizhu powder compound 256 active ingredients after de-duplication.

10.7717/peerj.13939/supp-3 Supplemental Information 3 All primers of RT-qPCR

10.7717/peerj.13939/supp-4 Supplemental Information 4 Molecular dynamics simulation for perspective 1 Pattern diagram of MDS changes of Rutin-AHR complex in aqueous solution.

10.7717/peerj.13939/supp-5 Supplemental Information 5 Molecular dynamics simulation for perspective 2 Pattern diagram of MDS changes of Rutin-AHR complex in aqueous solution.

## Additional Information and Declarations

### Data Availability

The following information was supplied regarding data availability: Raw data are available in the Supplemental Files.
