# Supplementary figures and images for: Prevention of polycystic ovary syndrome and postmenopausal osteoporosis by inhibiting apoptosis with Shenling Baizhu powder compound
Source: PeerJ. 2022 Oct 28;10:e13939. doi: 10.7717/peerj.13939 (PMC9620975; doi:10.7717/peerj.13939)

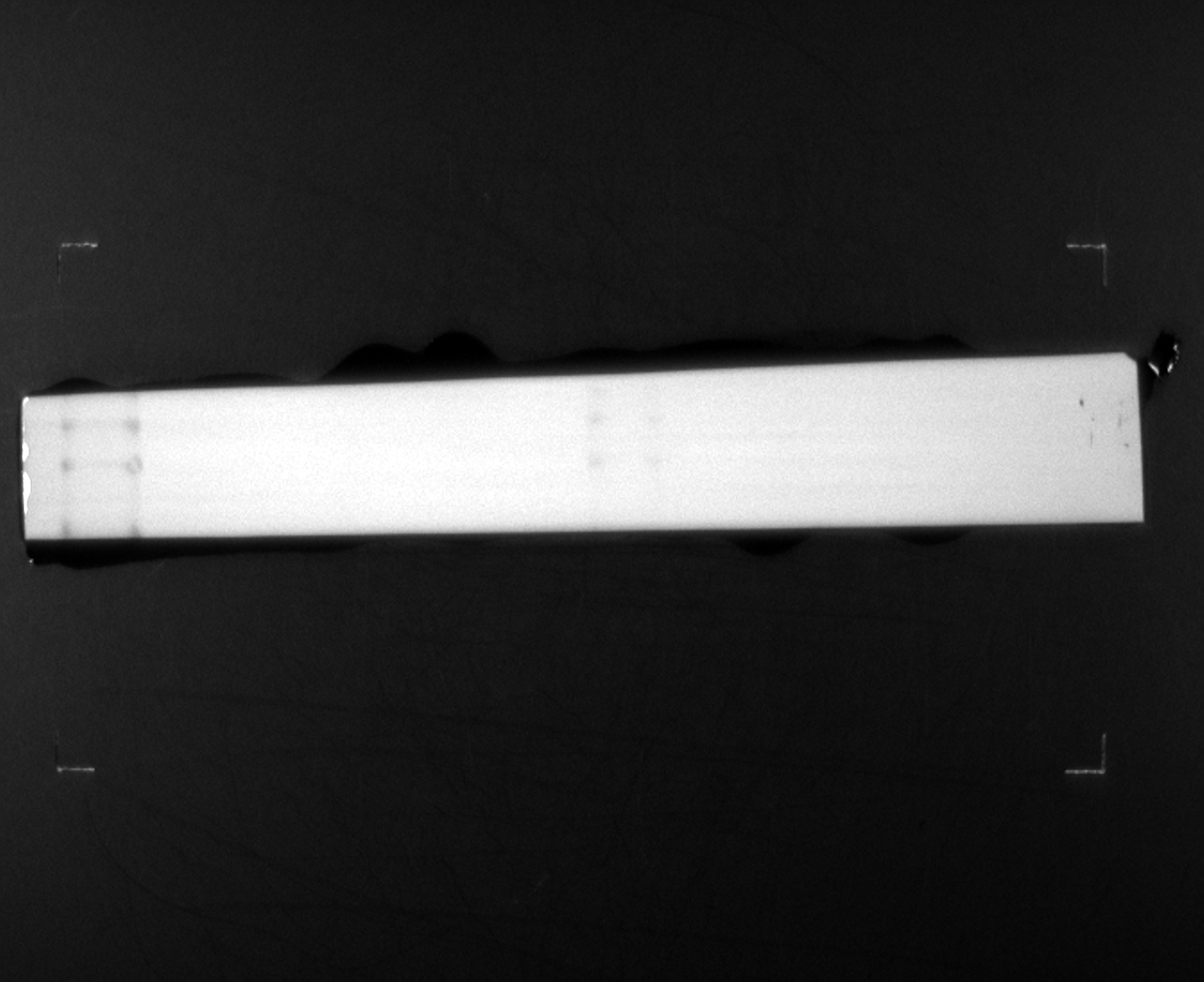

Supplement: Supplemental Information 1 [file peerj-10-13939-s001.zip › rawdate and plot/wb/5-AHR/0.Tif]

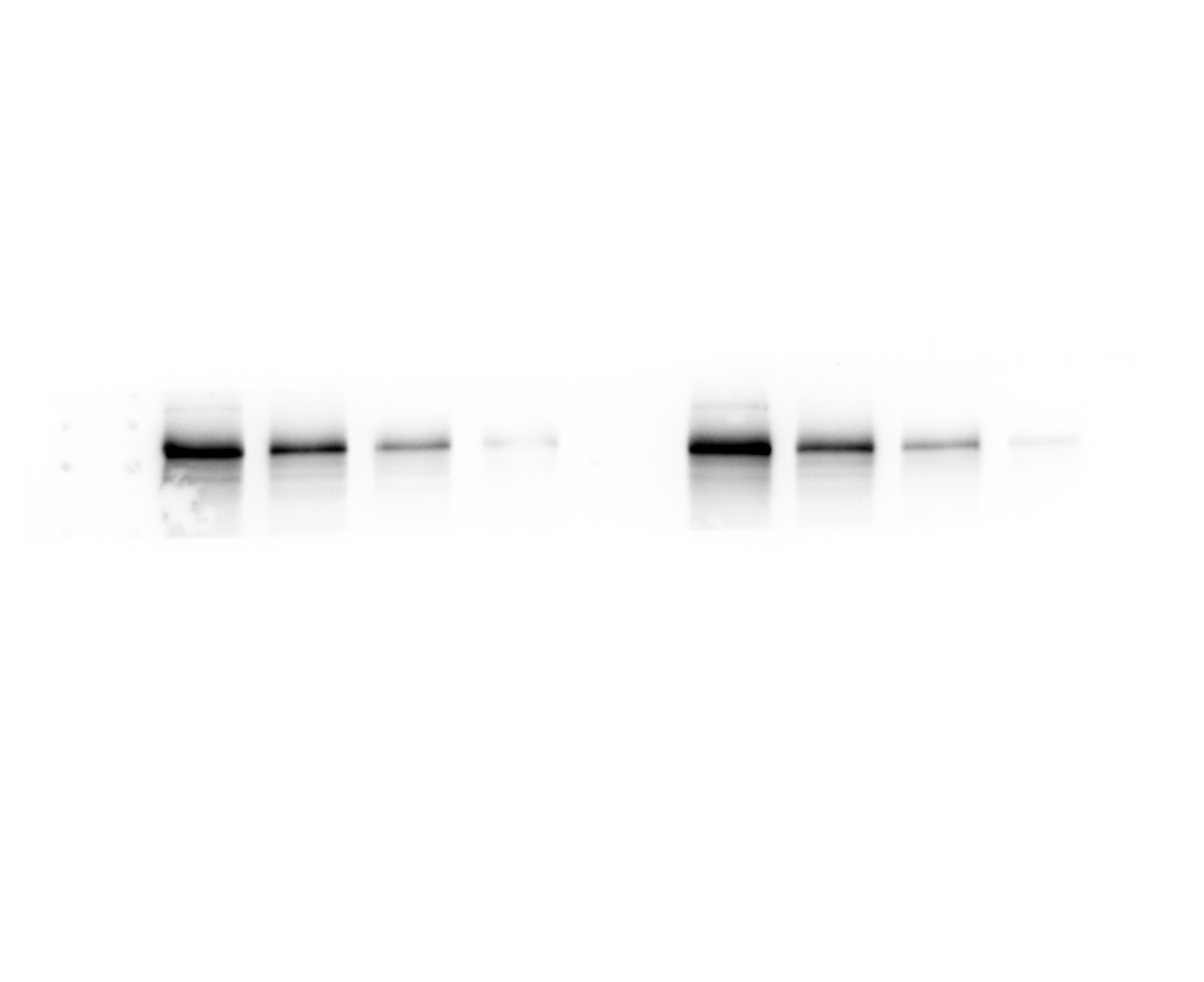

Supplement: Supplemental Information 1 [file peerj-10-13939-s001.zip › rawdate and plot/wb/5-AHR/10.Tif]

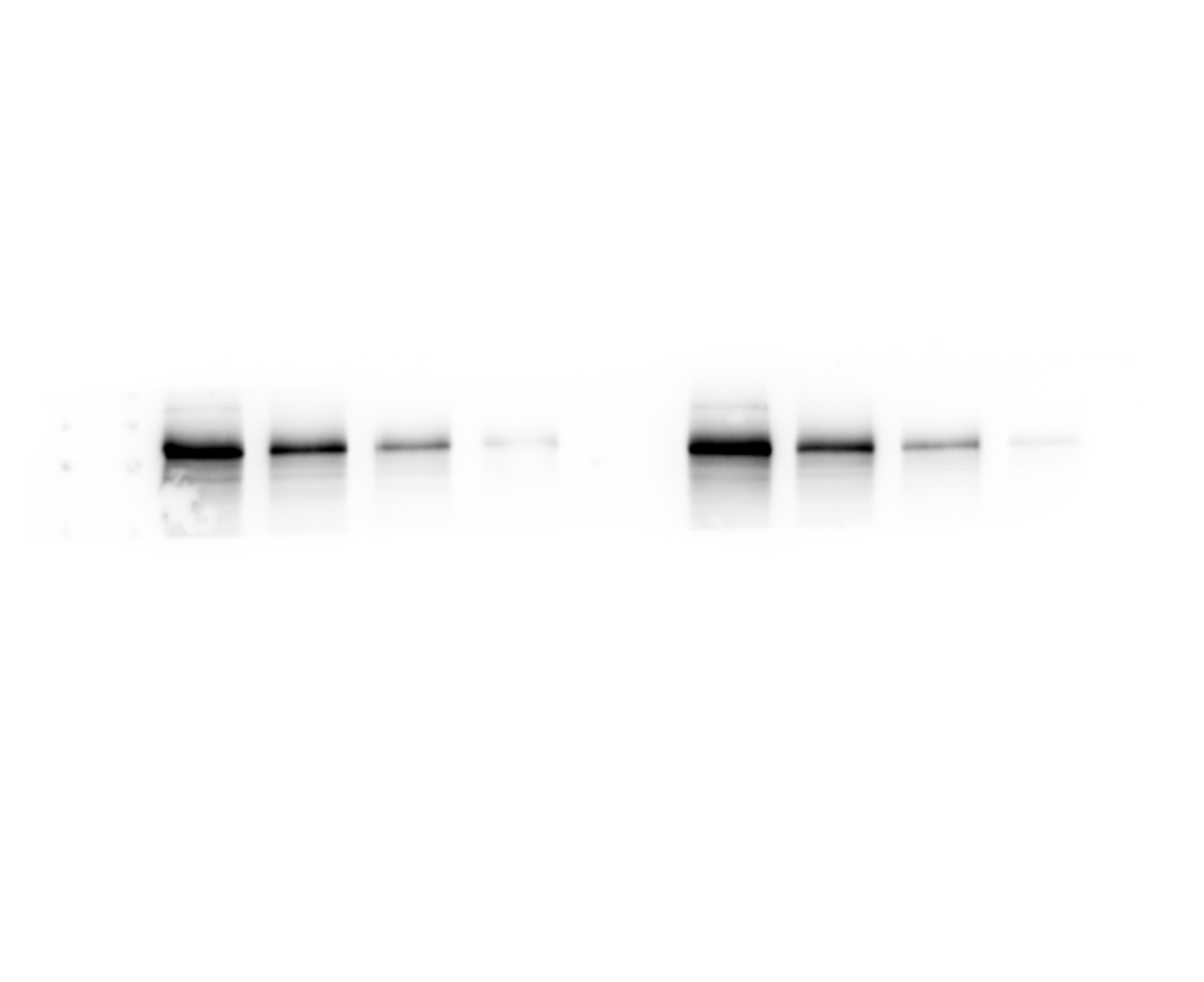

Supplement: Supplemental Information 1 [file peerj-10-13939-s001.zip › rawdate and plot/wb/5-AHR/20.Tif]

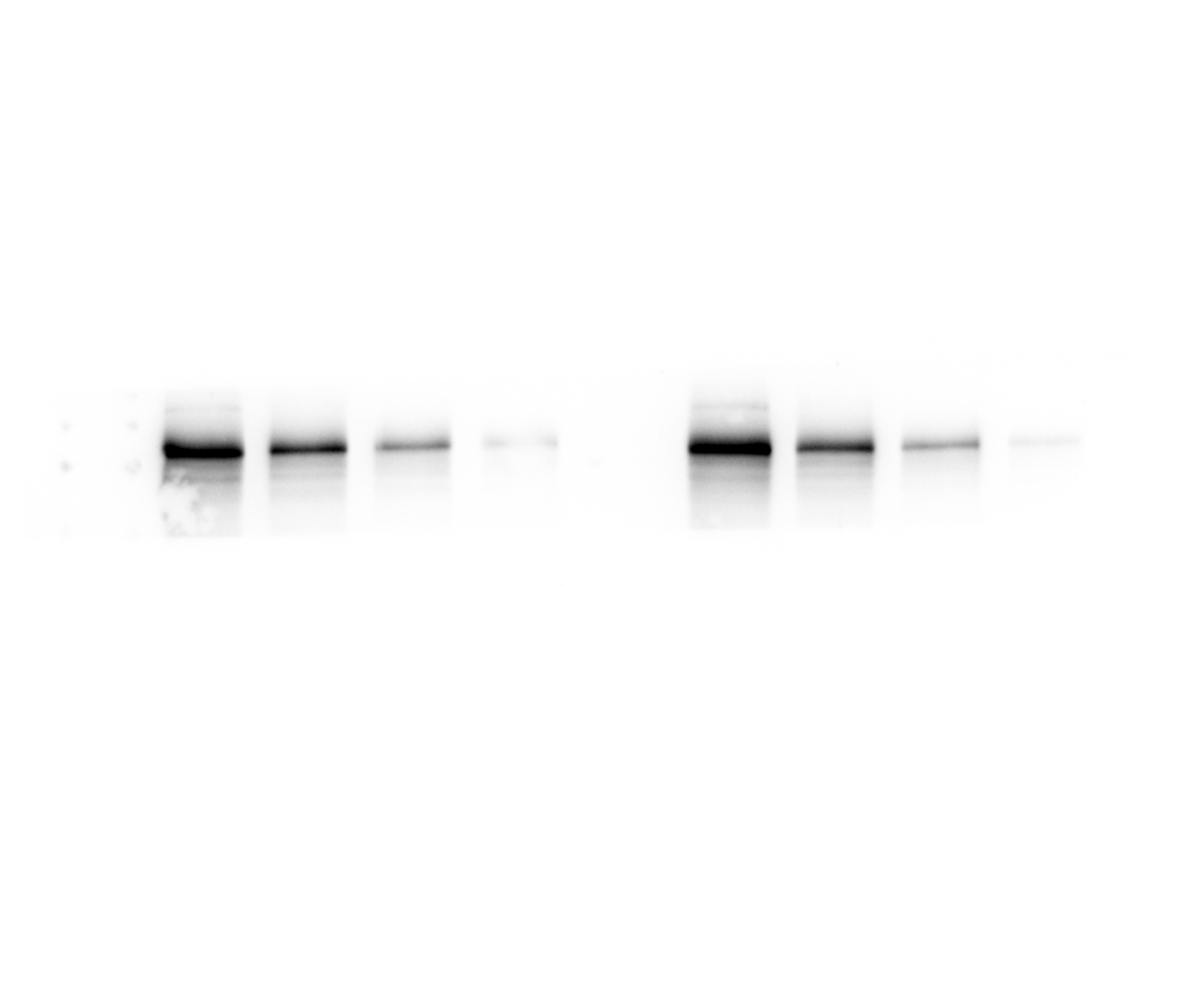

Supplement: Supplemental Information 1 [file peerj-10-13939-s001.zip › rawdate and plot/wb/5-AHR/7.Tif]

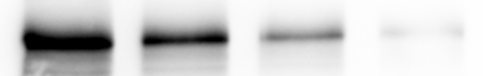

Supplement: Supplemental Information 1 [file peerj-10-13939-s001.zip › rawdate and plot/wb/5-AHR/Rutin-1 AHR.tif]

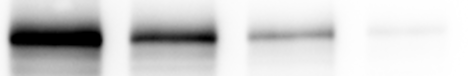

Supplement: Supplemental Information 1 [file peerj-10-13939-s001.zip › rawdate and plot/wb/5-AHR/Rutin-2 AHR.tif]

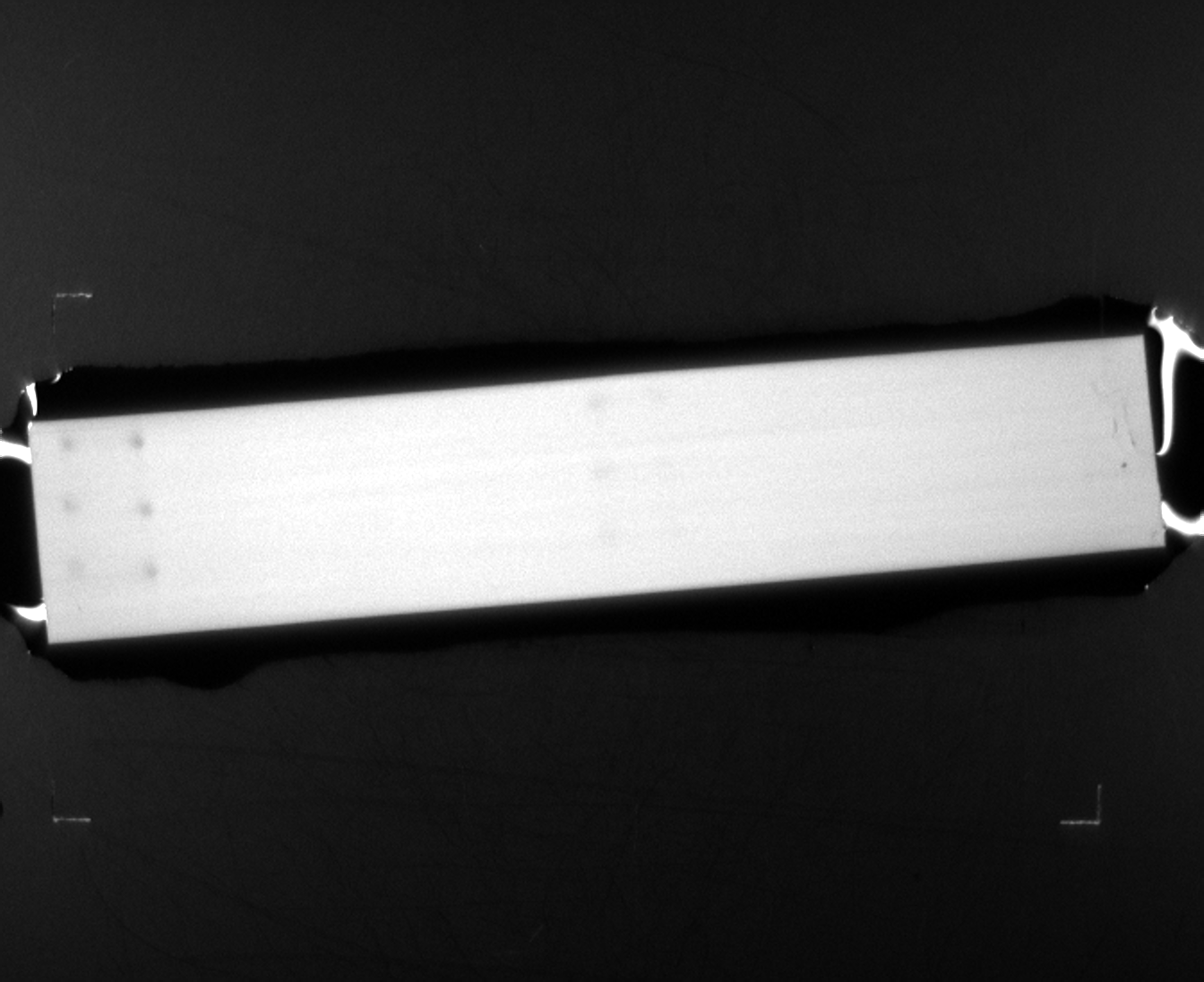

Supplement: Supplemental Information 1 [file peerj-10-13939-s001.zip › rawdate and plot/wb/5-C-3/0.Tif]

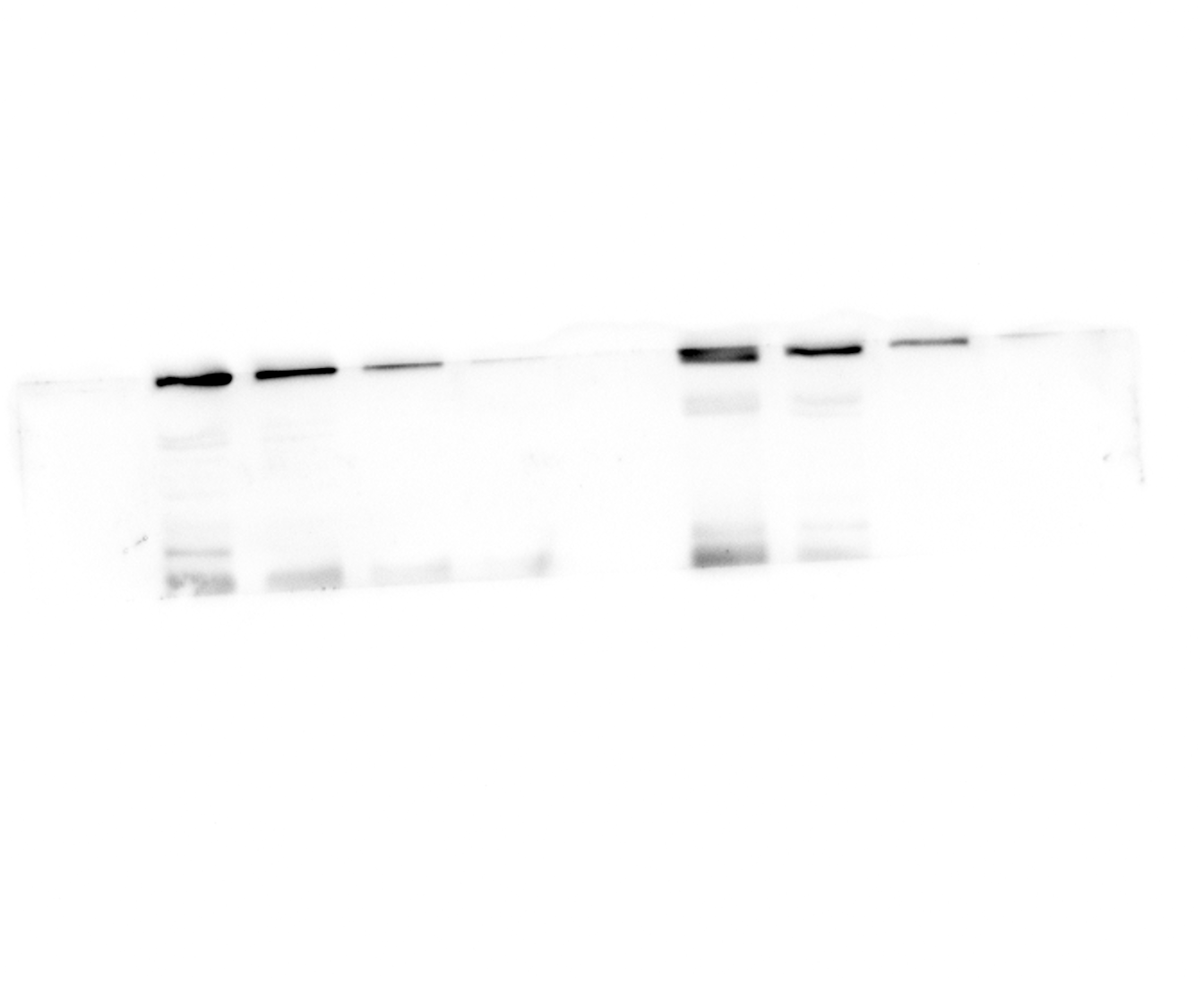

Supplement: Supplemental Information 1 [file peerj-10-13939-s001.zip › rawdate and plot/wb/5-C-3/24.Tif]

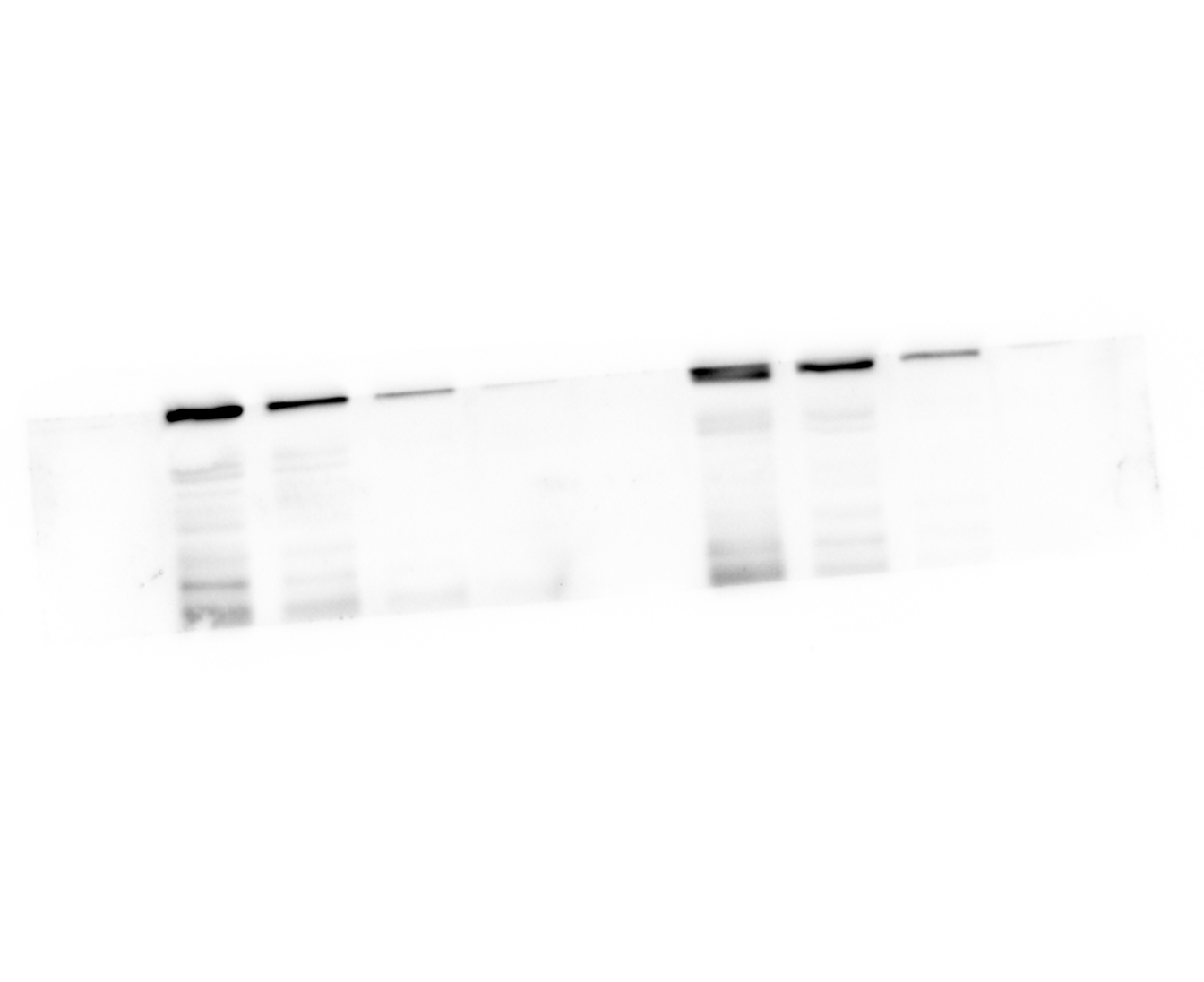

Supplement: Supplemental Information 1 [file peerj-10-13939-s001.zip › rawdate and plot/wb/5-C-3/30.Tif]

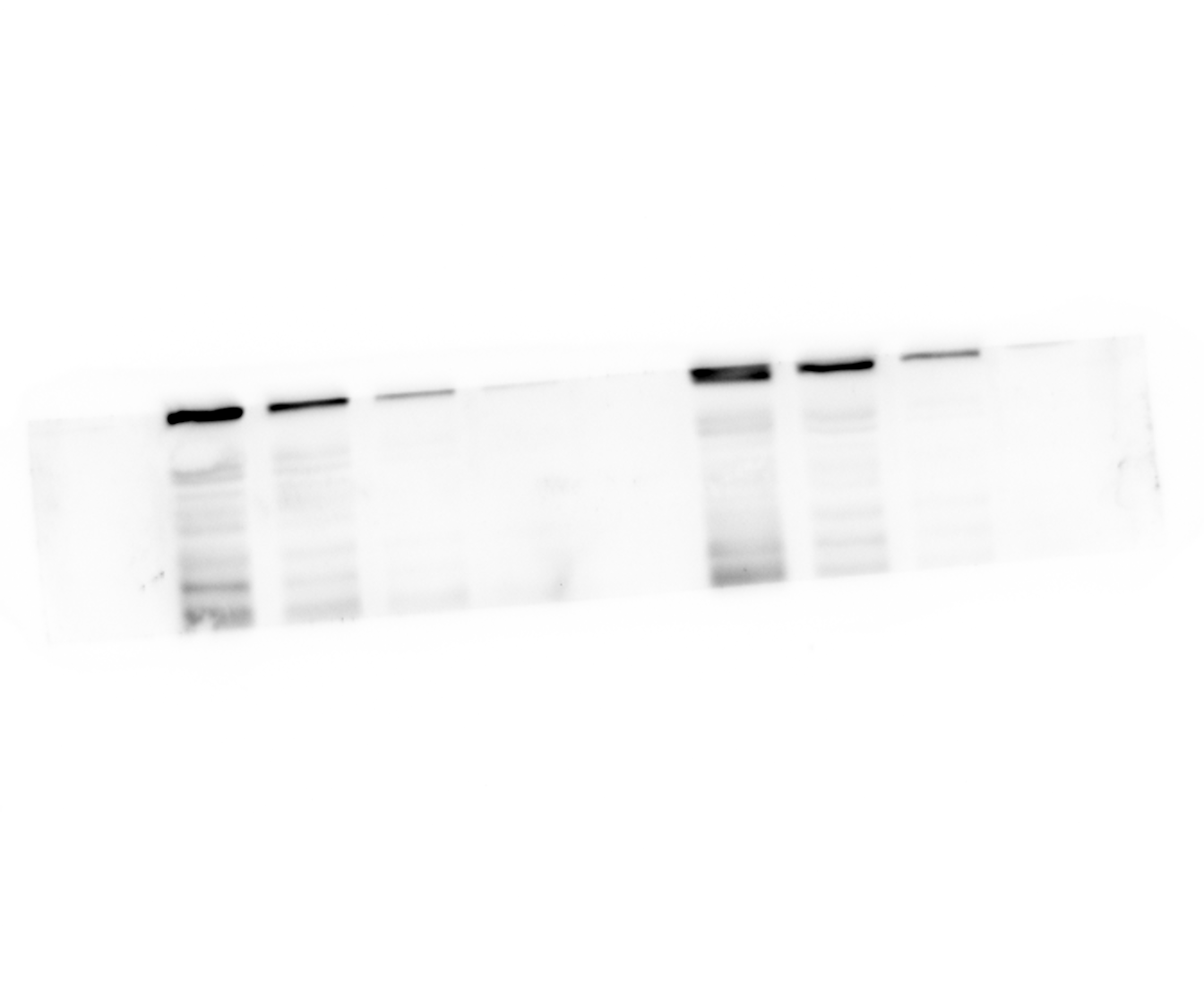

Supplement: Supplemental Information 1 [file peerj-10-13939-s001.zip › rawdate and plot/wb/5-C-3/50.Tif]

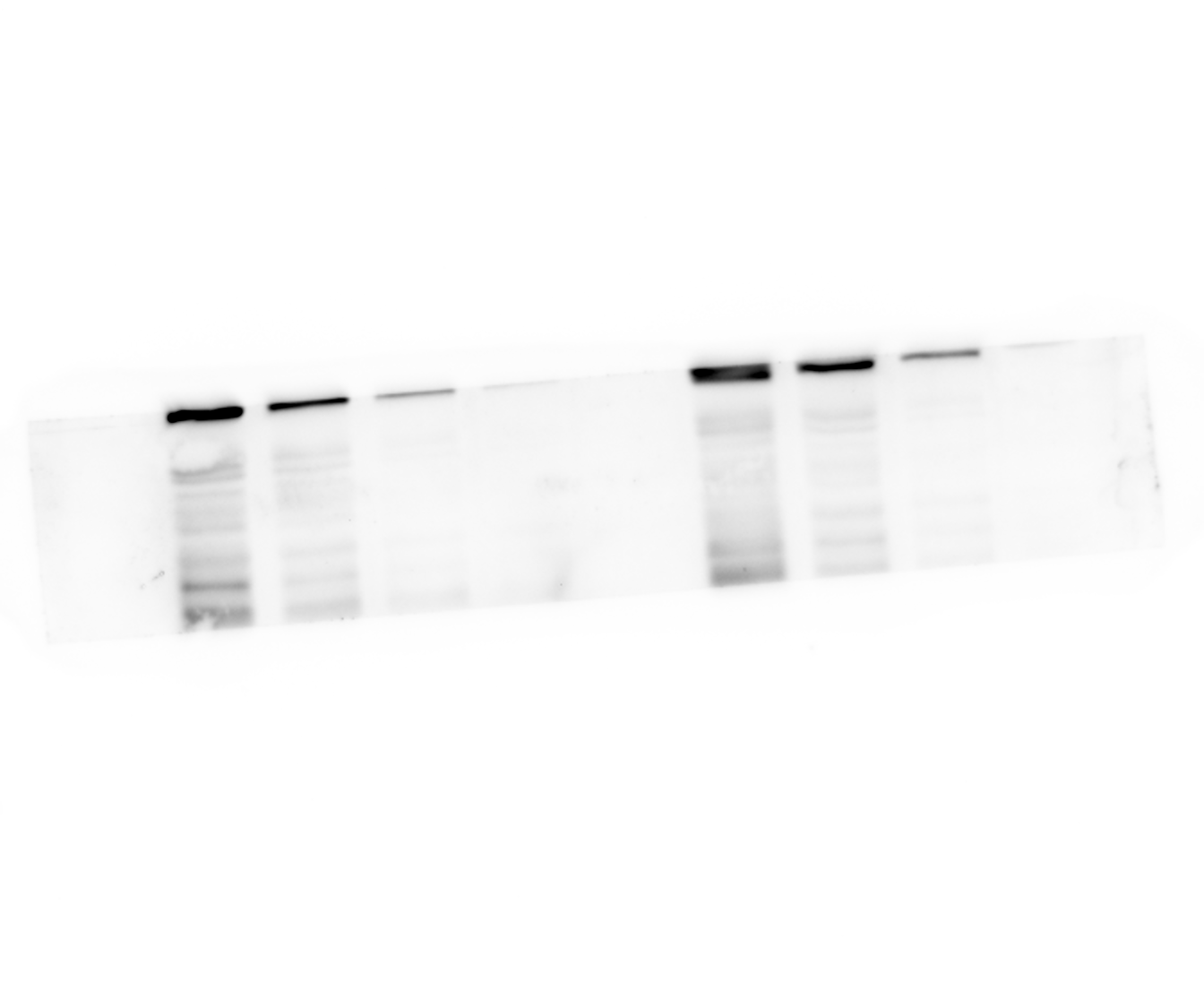

Supplement: Supplemental Information 1 [file peerj-10-13939-s001.zip › rawdate and plot/wb/5-C-3/80.Tif]

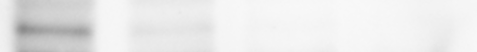

Supplement: Supplemental Information 1 [file peerj-10-13939-s001.zip › rawdate and plot/wb/5-C-3/Rutin-1 c-3.tif]

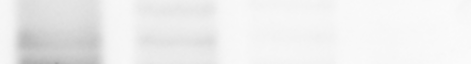

Supplement: Supplemental Information 1 [file peerj-10-13939-s001.zip › rawdate and plot/wb/5-C-3/Rutin-2 c-3.tif]

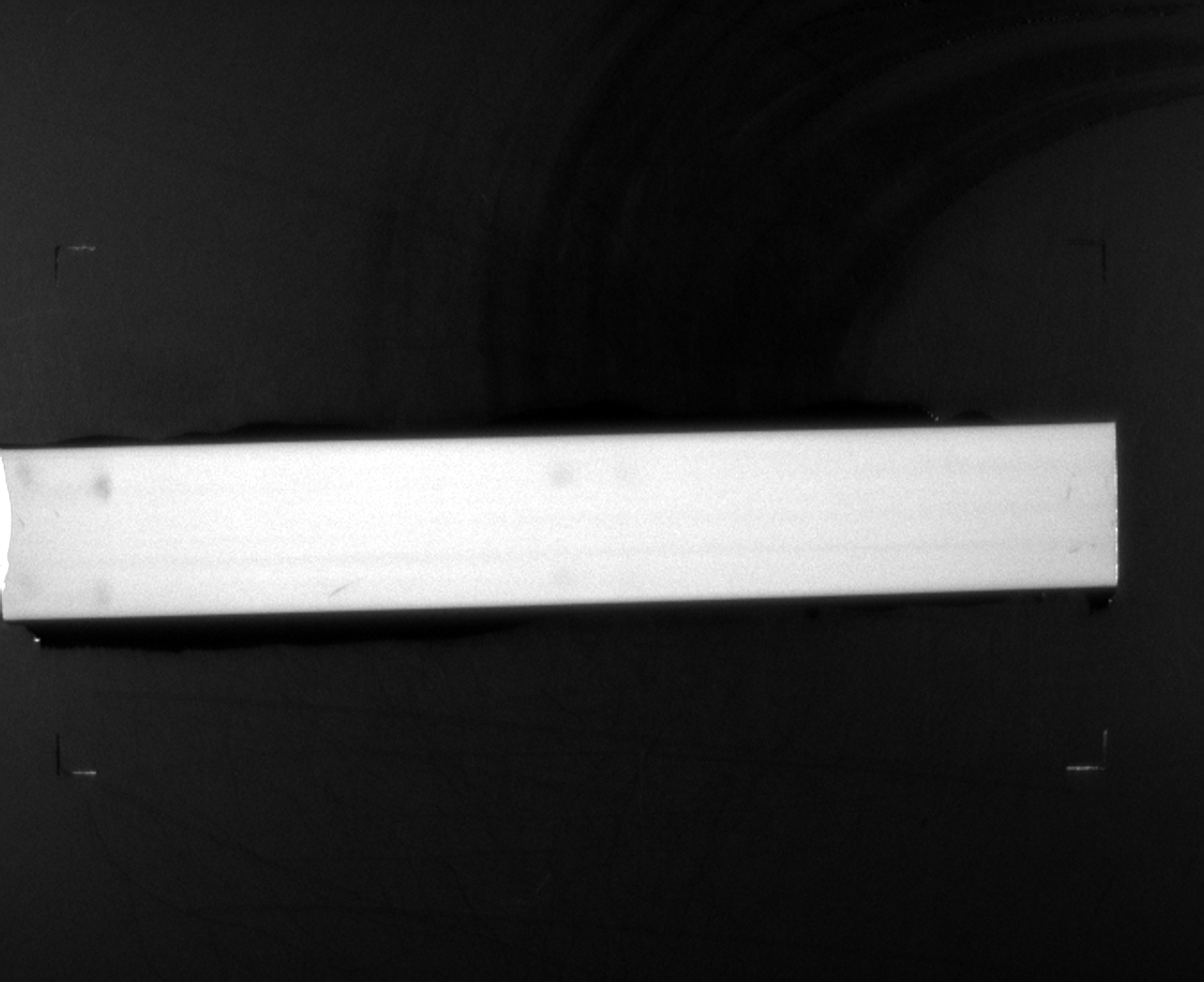

Supplement: Supplemental Information 1 [file peerj-10-13939-s001.zip › rawdate and plot/wb/5-C-C-3/0.Tif]

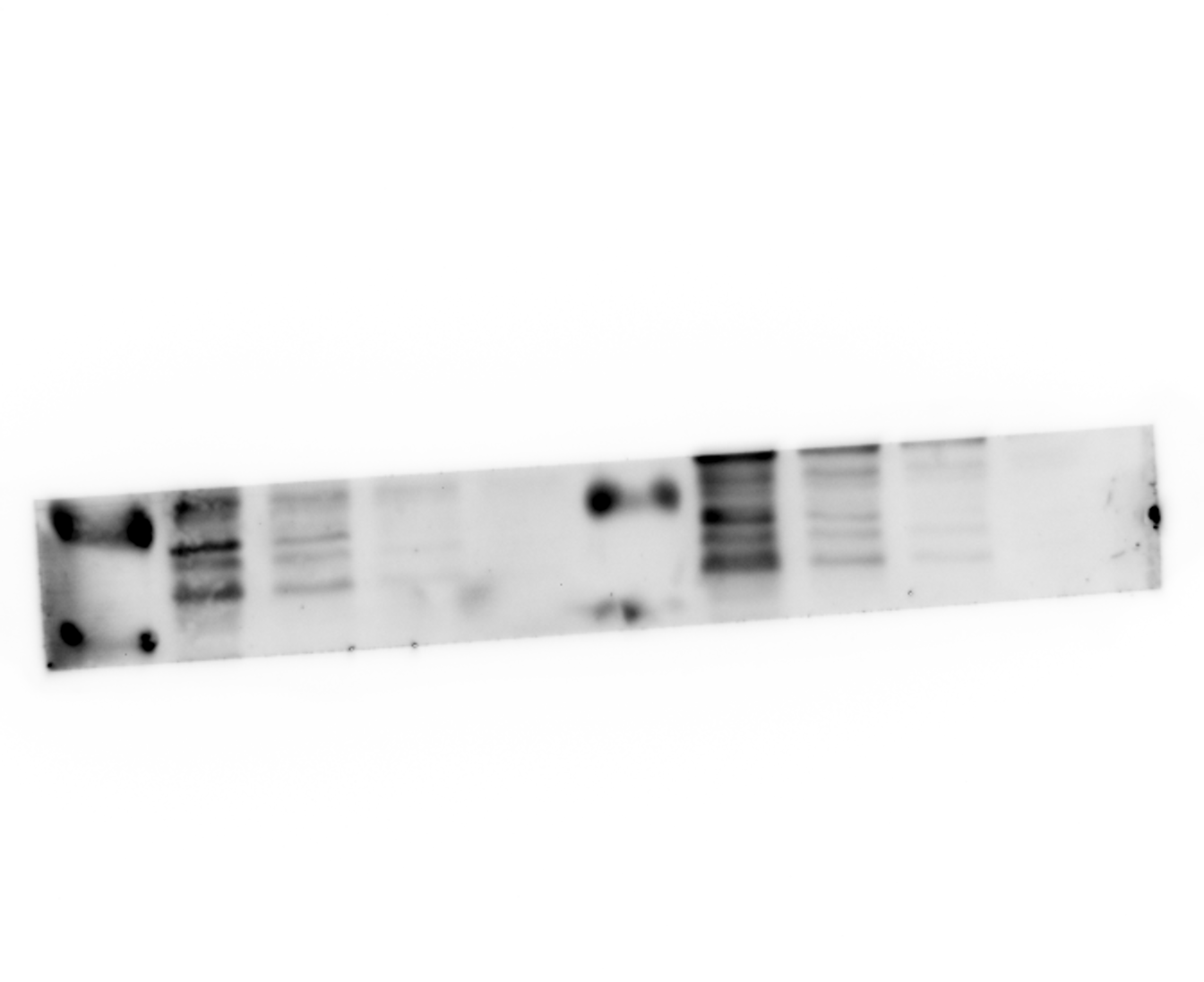

Supplement: Supplemental Information 1 [file peerj-10-13939-s001.zip › rawdate and plot/wb/5-C-C-3/150.Tif]

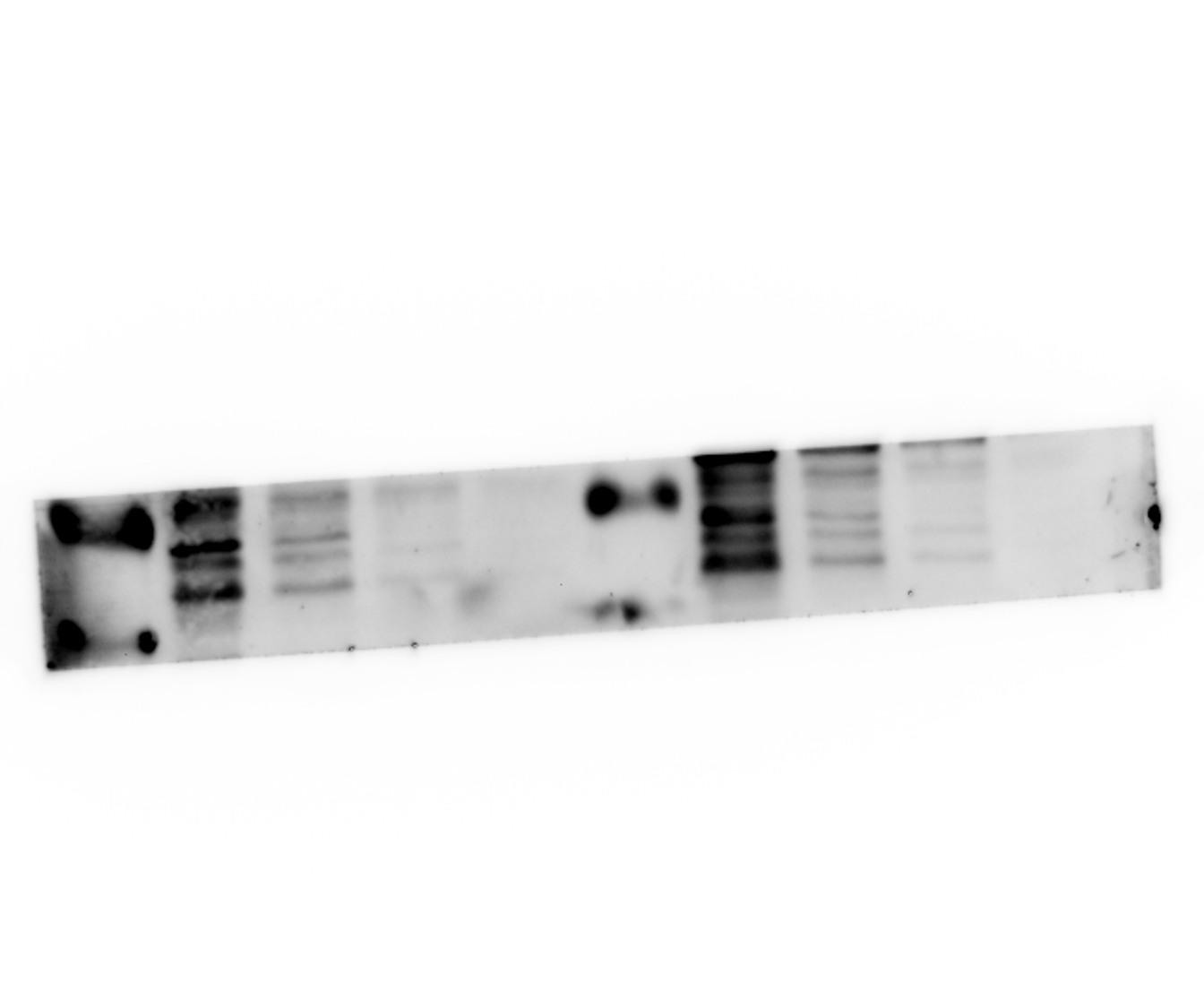

Supplement: Supplemental Information 1 [file peerj-10-13939-s001.zip › rawdate and plot/wb/5-C-C-3/200.Tif]

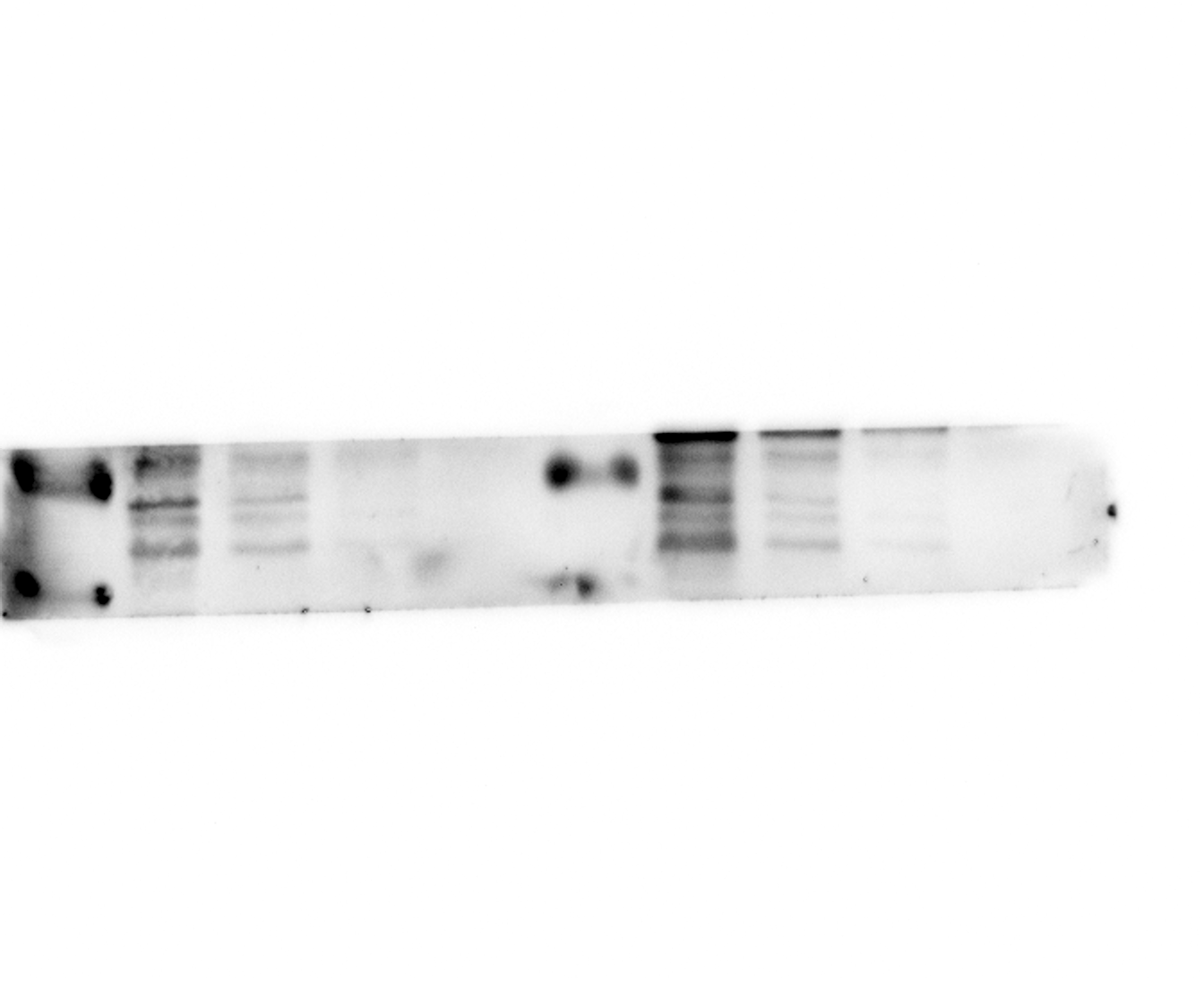

Supplement: Supplemental Information 1 [file peerj-10-13939-s001.zip › rawdate and plot/wb/5-C-C-3/26.Tif]

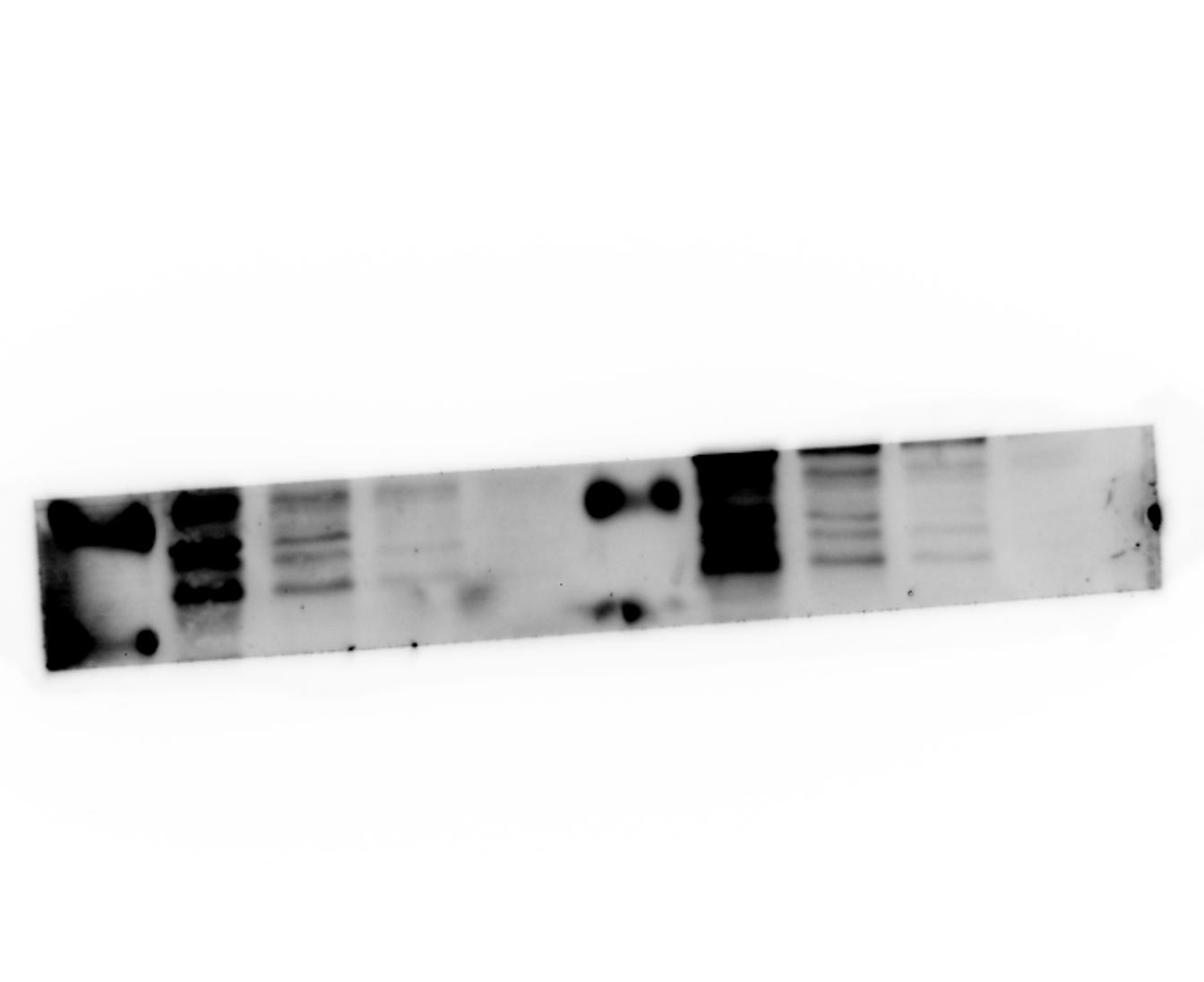

Supplement: Supplemental Information 1 [file peerj-10-13939-s001.zip › rawdate and plot/wb/5-C-C-3/300.Tif]

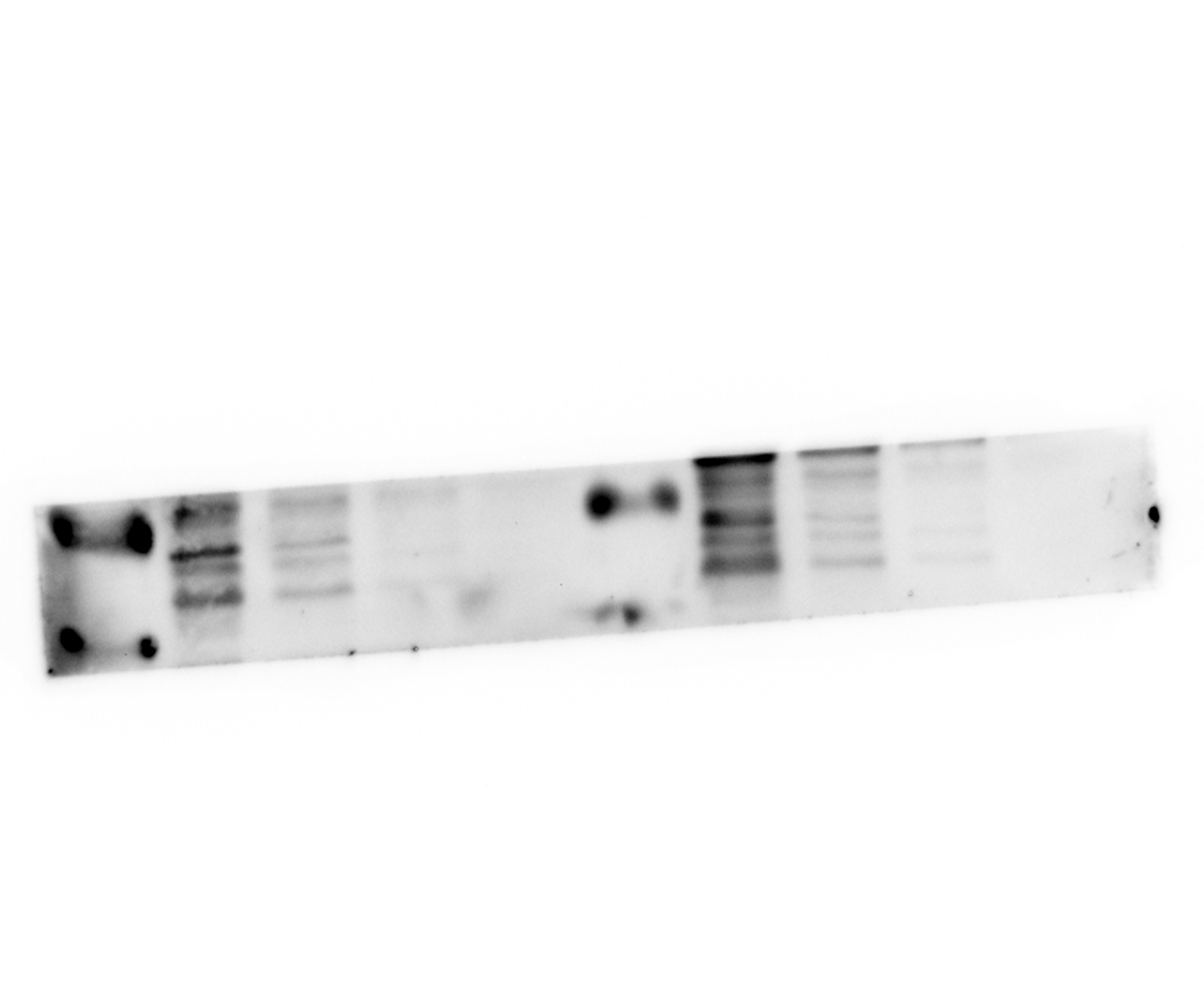

Supplement: Supplemental Information 1 [file peerj-10-13939-s001.zip › rawdate and plot/wb/5-C-C-3/50.Tif]

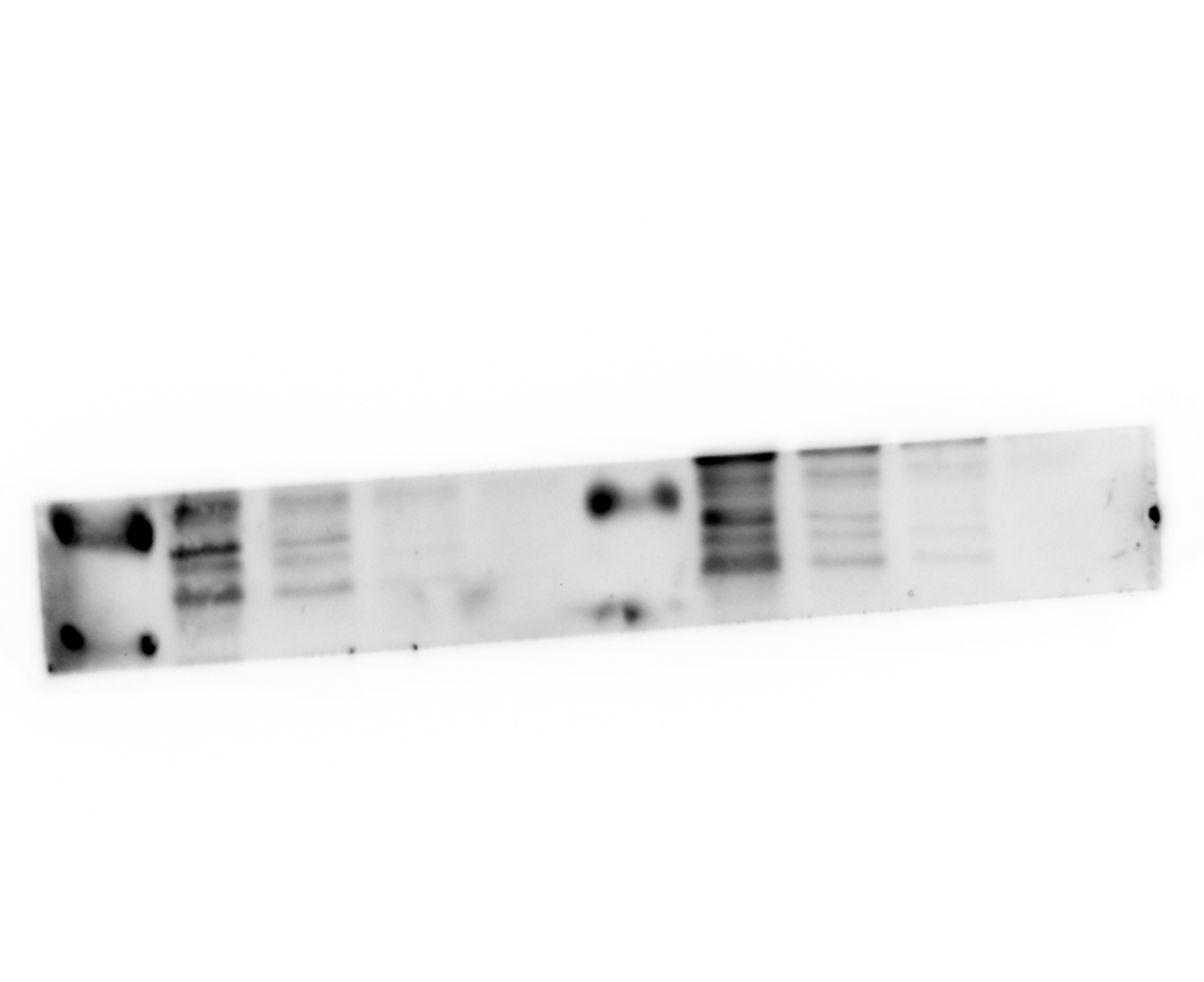

Supplement: Supplemental Information 1 [file peerj-10-13939-s001.zip › rawdate and plot/wb/5-C-C-3/80.Tif]

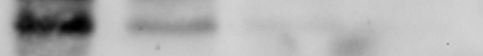

Supplement: Supplemental Information 1 [file peerj-10-13939-s001.zip › rawdate and plot/wb/5-C-C-3/Rutin-1 c-c-3.tif]

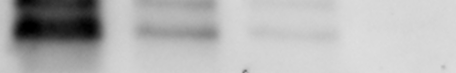

Supplement: Supplemental Information 1 [file peerj-10-13939-s001.zip › rawdate and plot/wb/5-C-C-3/Rutin-2 c-c-3.tif]

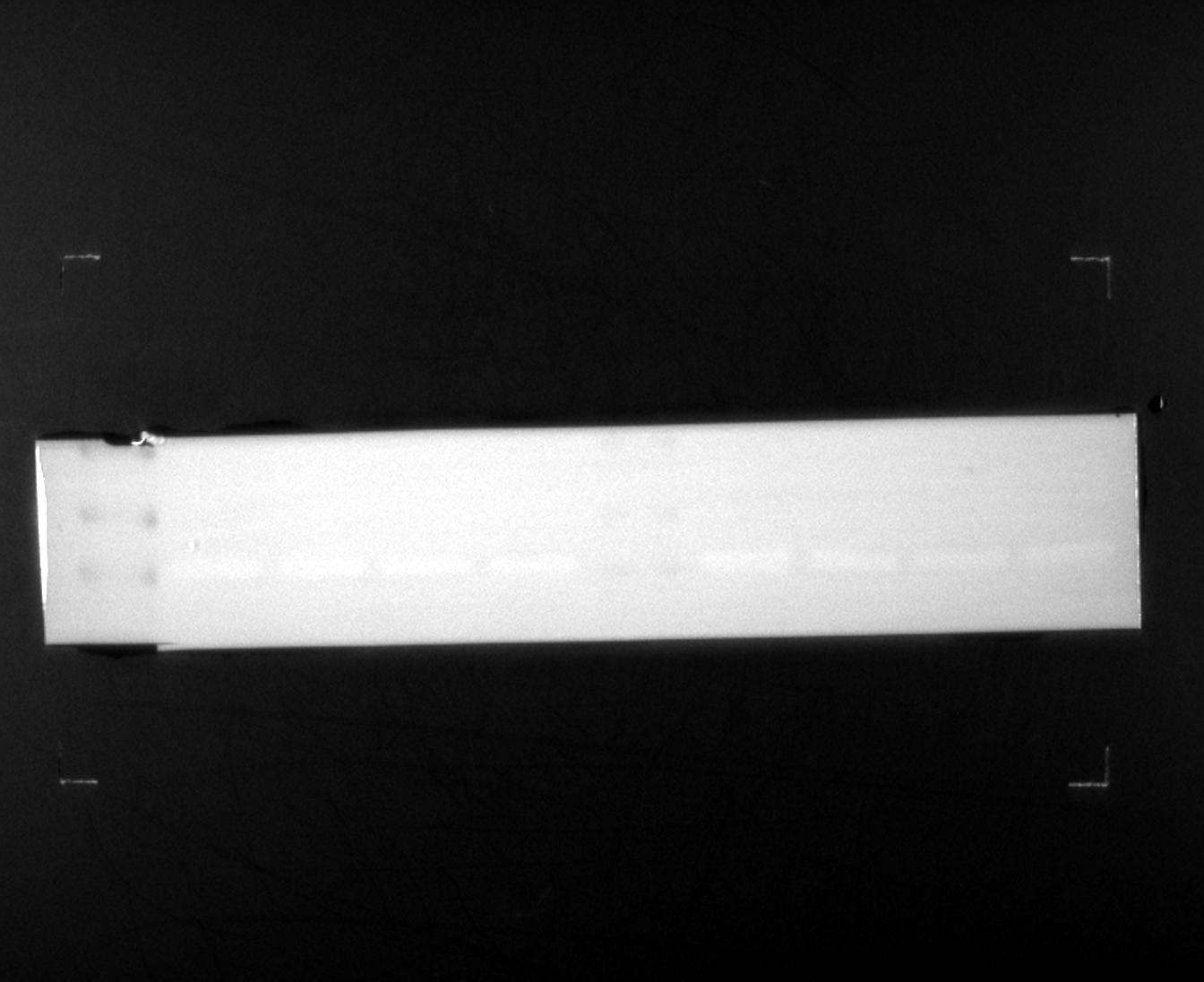

Supplement: Supplemental Information 1 [file peerj-10-13939-s001.zip › rawdate and plot/wb/6-GAPDH/0.Tif]

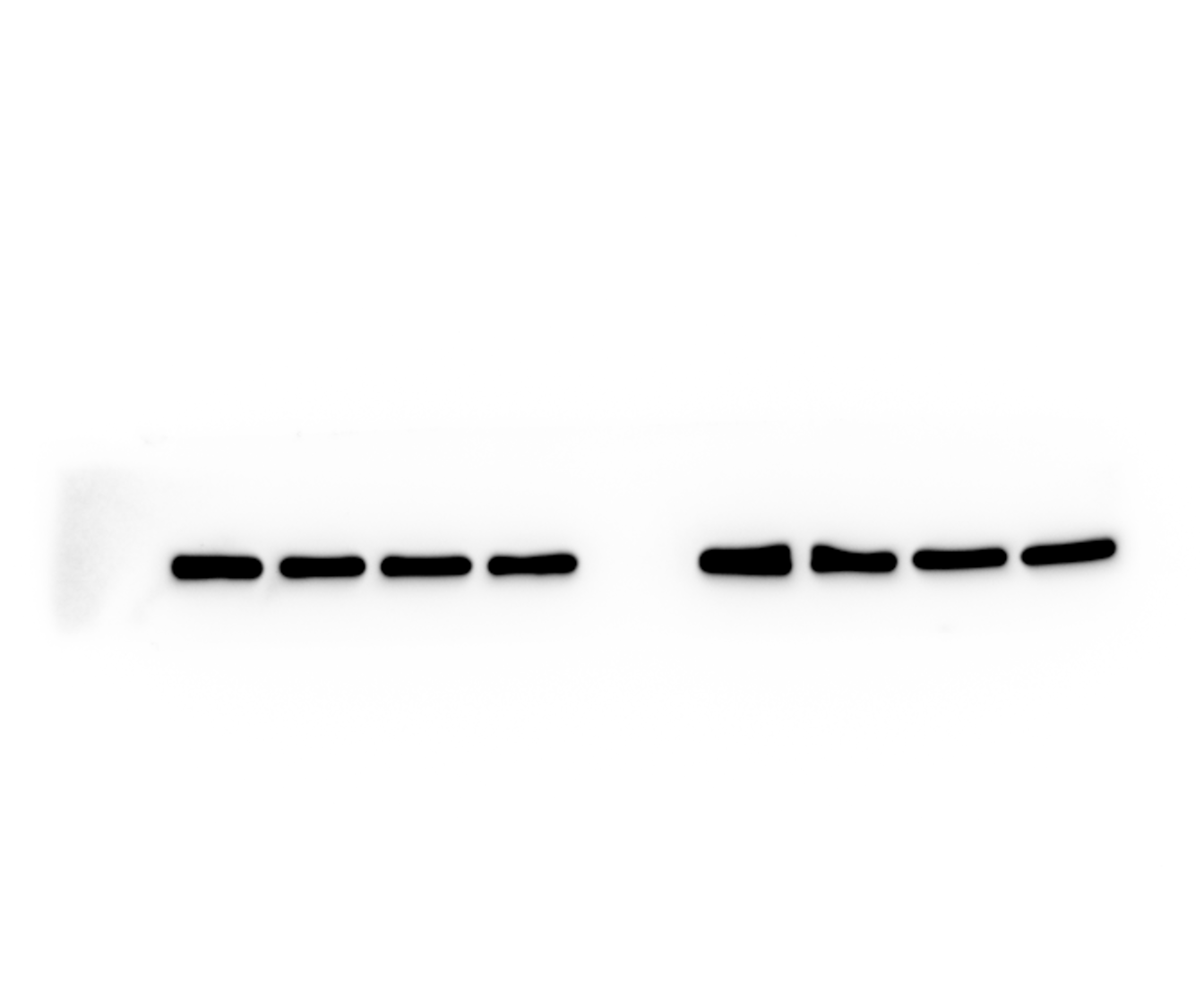

Supplement: Supplemental Information 1 [file peerj-10-13939-s001.zip › rawdate and plot/wb/6-GAPDH/10.Tif]

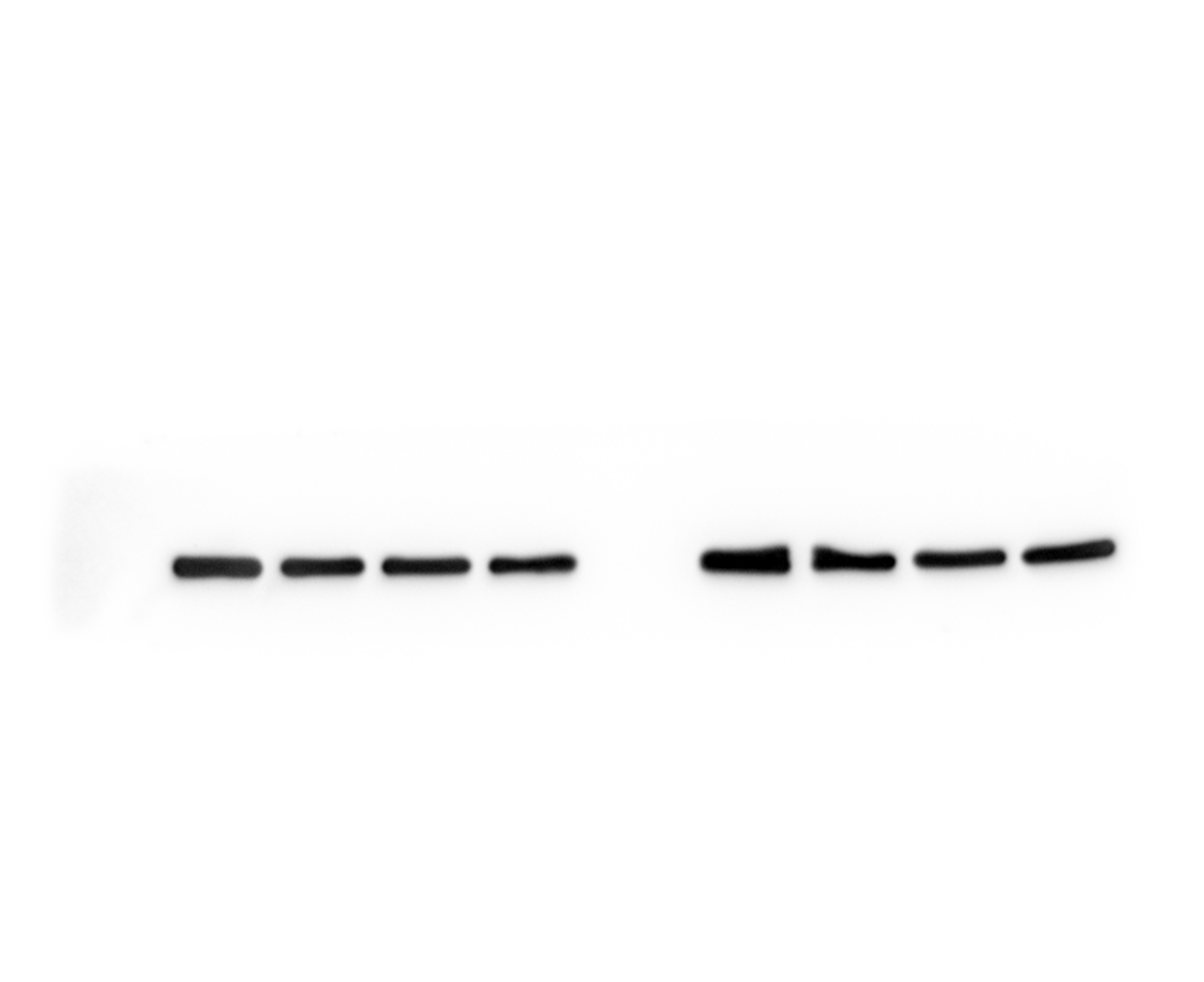

Supplement: Supplemental Information 1 [file peerj-10-13939-s001.zip › rawdate and plot/wb/6-GAPDH/2.2.Tif]

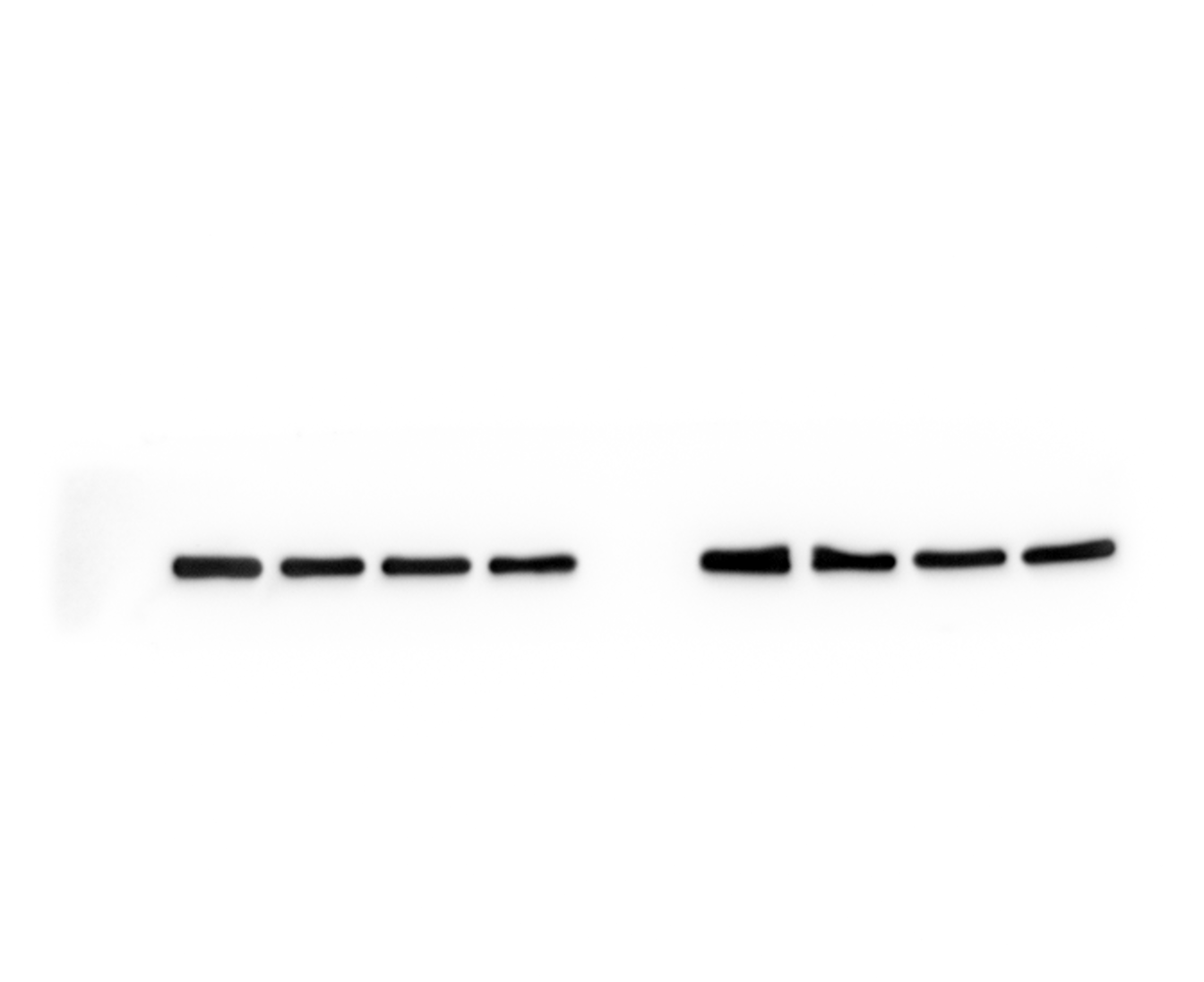

Supplement: Supplemental Information 1 [file peerj-10-13939-s001.zip › rawdate and plot/wb/6-GAPDH/3.Tif]

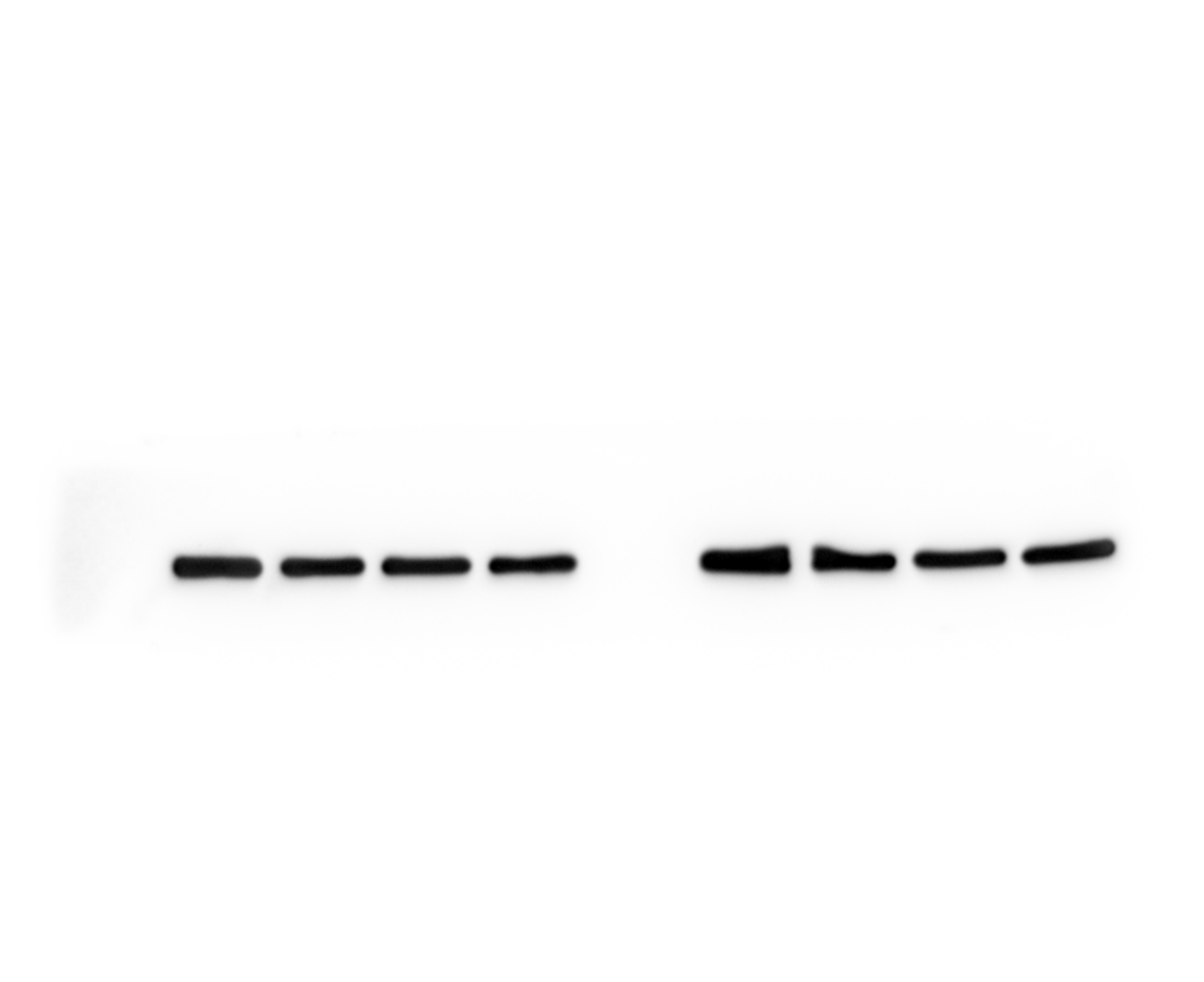

Supplement: Supplemental Information 1 [file peerj-10-13939-s001.zip › rawdate and plot/wb/6-GAPDH/5.Tif]

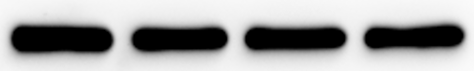

Supplement: Supplemental Information 1 [file peerj-10-13939-s001.zip › rawdate and plot/wb/6-GAPDH/Rutin-1 GAPDH.tif]

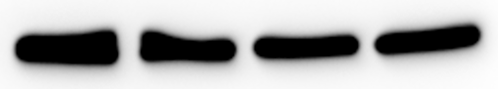

Supplement: Supplemental Information 1 [file peerj-10-13939-s001.zip › rawdate and plot/wb/6-GAPDH/Rutin-2 GAPDH.tif]
